# Investigation on improving immunologic reconstitution insufficiency using DiwuYanggan capsules in AIDS patients

**DOI:** 10.3389/fphar.2024.1485719

**Published:** 2024-11-06

**Authors:** Jing Wen Ke, Yao Chen, En Ze Lei, Ming Zhong Xiao, Wei Ni, Fang Huang, Han Min Li, Hong Lin Jiang, Lian Guo Ruan, Jian Zhong Liu

**Affiliations:** ^1^ Hubei Provincial Hospital of Traditional Chinese Medicine, Affiliated Hospital of Hubei University of Chinese Medicine, Wuhan, China; ^2^ Hubei Shizhen Laboratory, Wuhan, China; ^3^ Hubei University of Chinese Medicine, Wuhan, China; ^4^ Hubei Key Laboratory of Liver and Kidney Research and Application of Traditional Chinese Medicine, Wuhan, China; ^5^ Hubei Jiangxia Laboratory, Wuhan, Hubei, China; ^6^ Hubei provincial center for disease control and prevention, Wuhan, China; ^7^ Wuhan Jinyintan Hospital, Wuhan, China

**Keywords:** DiwuYanggan capsule, immunological non-responder, component analysis, network pharmacology, toll-like receptor signaling pathway

## Abstract

**Background:**

This study aimed to explore the mechanism of action of DiWuYangGan (DWYG) capsule in improving Immunological non-responder (INR) by analyzing the active ingredients of DWYG.

**Methods:**

The study employed a randomized, controlled, double-blind, single-simulation method. Patients were randomly divided into control and trial groups and treated with the primal highly effective antiretroviral therapy. To demonstrate the effect of DWYG on INR, patients in the control group were administered simulated DWYG, whereas patients in the trial group were administered DWYG capsules (ChiCTR1900024673). The chemical composition of DWYG was analyzed using ultra-performance liquid chromatography-high-resolution mass spectrometry. Potential targets of DWYG in the treatment of INR were identified and predicted using network pharmacology and molecular docking. The molecular mechanisms underlying the effects of DWYG were validated using a peripheral blood monocyte model.

**Results:**

The CD4:CD8 ratio in the trial group was significantly higher than that in the control group (*p* < 0.01). A total of 210 DWYG compounds were identified and network pharmacology revealed 182 potential therapeutic targets for DWYG and INR. The results of Gene Ontology and Kyoto Encyclopedia of Genes and Genomes analyses showed that the toll-like receptor signaling pathway is one of the key pathways. This study demonstrated that DWYG reduced the expression level of TLR4 and the levels of IL-2, IL-10, and TNF-α, which are important cytokines involved in the immune response.

**Conclusion:**

The efficacy of DWYG in the treatment of INR confirmed the potential practical components of DWYG. Moreover, the results of network pharmacology and experimental validation showed that DWYG could restore the immune function of acquired immune deficiency syndrome patients by inhibiting the expression of TLR4 and related signaling pathways and the overactivation of immune function.

**Clinical Trial Registration:**

https://www.chictr.org.cn/index.html, identifier ChiCTR1900024673.

## 1 Introduction

Immunological reconstitution insufficiency is a significant challenge in the treatment of acquired immune deficiency syndrome (AIDS). Highly effective antiretroviral therapy (HAART) can suppress human immunodeficiency virus (HIV) replication and reduce T-cell death, which improves the patient’s immune function. Despite receiving HAART for a certain period, some patients still have fewer CD4^+^T lymphocytes and are considered immunological non-responders (INRs) (Rb-Silva et al., 2019). Maintaining a stable viral load while restoring the immunologic function of INRs is a challenge in patients with AIDS and has been widely investigated. Numerous studies have reported that Chinese herbal medicines have the potential to improve immune function and treat Immunological non-responder (INR) (Ji et al., 2022).

However, the mechanism of action of INR remains unclear. Numerous factors, such as age, sex, HAART treatment duration, and body mass index, affect the immunological reconstitution of INRs (Lei et al., 2022; Yang et al., 2020). Low initial CD4^+^T lymphocytes, older age found to be significant risk factors for INR. (Roul et al., 2018; Zhang et al., 2020). Currently, some evidence suggests that CD4^+^T lymphocytes are excessively depleted owing to the abnormal activation of immunological functions, which accelerates the renewal of T cells (Sokoya et al., 2017). Monocytes, which are key components of innate immunity, play an important role in the recovery of immunological functions in INRs. Abnormal monocytes may produce cytokines that trigger local or systemic inflammation, leading to the hyperactivity of immunological functions (Anzinger et al., 2014). Toll-like receptors (TLR) are a subfamily of important pattern-recognition receptors in the innate immune system. TLR can control inflammasome activity and induce the release of cytokines to regulate inflammatory responses and maintain immune balance. Simultaneously, TLR can induce immune cells to express costimulatory molecules and promote the activation and proliferation of T cells (Brubaker et al., 2015; Fitzgerald and Kagan, 2020).

Previous studies have confirmed that DiWuYangGan (DWYG) can promote the proliferation and differentiation of bone marrow stem cells and regulate the secretion of related immune cytokines. Thus, DWYG has a bidirectional regulatory effect on the states of overactivation and hypofunction of immunologic function (Zhang et al., 2023; Xu et al., 2020). According to our previous clinical study conducted for 24 weeks, DWYG could effectively increase the proportion of CD45RA^+^ in INRs to promote immunologic reconstitution and had good clinical safety (Lei et al., 2022). However, the mechanism underlying the effects of DWYG on INR remains unclear; therefore, this study aimed to study its mechanism of action and lay the foundation for further expansion of its clinical application.

## 2 Materials and methods

### 2.1 Preparation of DWYG

DWYG (E Yao Zhi Zi: Z20113160) is mainly composed of Radix Rehmanniae Praeparata (*Rehmannia glutinosa*, SDH), Chinese Magnolcavine Fruit (*Schisandrae Chinensis Fructus*, WWZ), Virgate Wormwood Herb (*Artemisiae Scopariae Herba*, YC), turmeric (*Curcumae Longae Rhizoma*, JH), and licorice roots of north-western origin (*Radix Glycyrrhizae*, SGC). Simulated DWYG (1/20 of the original DWYG with coloring excipients and flavor excipients). DWYG and simulated DWYG were obtained from the manufacturing laboratory of Hubei Provincial Hospital of Traditional Chinese Medicine.

### 2.2 Controlled clinical trials

Patients with HIV/AIDS who visited Wuhan Jinyintan Hospital between December 2019 and March 2021 were selected for this clinical trial. The trial included individuals who met the following inclusion criteria. 1) Patients should meet the diagnostic criteria for AIDS-INR. 2) Patients must be between 18 and 65 years old. 3) Patients must the belong to the yin deficiency type in the liver and kidney through TCM syndrome differentiation. 4) Patients must sign the informed consent form and thus can be followed up on time.

The exclusion criteria were as follows. 1) Serious opportunistic infections in patients are not controlled before enrollment. 2) Patients that have multi-system diseases. 3) Patients with tumors or allergies 4) Patients who do not fully understand the trial or cooperate well. 5) The investigator considered the patients unfit to participate in the trial in certain cases.

This controlled clinical trial was approved by the Ethics Committee of Hubei Hospital of Traditional Chinese Medicine (No. HBZY2018-C23-01).

The present study employed a randomized, controlled, double-blind, single-simulation method. The study registration number of the Chinese Clinical Trial Registry (ChiCTR) is ChiCTR1900024673. SPSS 19.0 software was used to divide the patients who met the inclusion and exclusion criteria into two groups according to a random number table and using the HAART medication regimen. Patients in the control group were administered simulated DWYG, whereas those in the trial group were administered DWYG. The dosage is 3 times a day, 5 capsules each time.

The ratio of CD4^+^ to CD8^+^T cells in peripheral blood at baseline and after 24 weeks of treatment were observed and recorded. The samples were examined using flow cytometry (BD FACSCanto II). The proportion of T lymphocyte subsets was determined using Diva 8.0.2 Software. The reagents used included CD4 FITC/CD8 PE (Becton, Dickinson and Company).

The SAS 9.1 software was used for Statistical analysis. Normally distributed measurement data are expressed as mean ± SD, and skewed distribution data are expressed as M (P_25_, P_75_). Baseline data were compared between the groups using the *t*-test, and 24 week data were compared between the groups using the Mann-Whitney rank-sum test. Differences between 24 weeks and baseline were compared between groups using the Wilcoxon rank-sum test. All statistical tests were two-sided, and *p* < 0.05 was considered statistically significant.

### 2.3 Identification of active ingredients in DWYG ultra-performance liquid chromatography-high resolution mass spectrometry (UPLC-Q-TOF/MS)

Waters Xevo G2-XS QTOF, ACQUITY UPLC M-Class, Waters ACQUITY REH C18 (1.7 μm, 2.1 × 100 mm). The following chromatographic parameters were used: column temperature, 40°C; mobile phase A, 0.1% formic acid in water; and mobile phase B, methanol. Gradient elution was employed with the following specifications: 0 min, 10% B; 15 min, 55% B; 35 min, 90% B; 40 min, 98% B; 45 min, 98% B; 46 min, 10% B; and 50 min, 10% B. The flow velocity was set at 0.3 mL/min, and the sample size was 2 μL.

The mass spectrometry conditions were as follows: ESI ion source was used to collect data in positive ion mode; primary and secondary mass spectrometry ions were collected in MSE mode, and the collection range was m/z 50–1,500 Da, cone voltage was 60 V, electrospray voltage was 3000 V, and the ESI ion source temperature was 100°C. The desolvation temperature was 500°C, the curtain gas flow rate was 50 L/h, and the desolvation gas (N_2_) flow rate was 600 L/h. The primary collision energy was 10 eV, the secondary collision energy was 35 eV, the collision energy fluctuation was ±10 eV, and an enkephalin (m/z 556.2771) tuning solution was employed as the real-time calibration solution. Based on the liquid-phase retention time, accurate molecular weight, mass spectrometry, and secondary fragmentation (Shi et al., 2023).

### 2.4 Network pharmacology analysis

The identified compounds were imported into the traditional Chinese medicine systems pharmacology database and analysis platform (TCMSP) (Ru et al., 2014) and Swiss Target Prediction (Gfeller et al., 2014) databases to predict the targets of the active ingredients based on the probability criterion (probability >0). The keyword “INR” was searched in the Gene Cards databases (Safran et al., 2010), and the therapeutic targets associated with INR were summarized. A Venn diagram was constructed to visualize the intersection of the compounds and disease targets. Genes intersecting the Venn diagram were imported into the STRING database (Szklarczyk et al., 2023) for protein-protein interaction (PPI) analysis. The STRING database was then imported into Cytoscape for topological analysis. CytoNCA (Tang et al., 2015) was used to perform the calculations, and R was employed to filter the disease targets according to the criteria of the two conditions for the analysis of core targets in the PPI. Gene Ontology (GO) and Kyoto Encyclopedia of Genes and Genomes (KEGG) (Kanehisa and Goto, 2000) analyses were performed using the ClusterProfile R package (Xu et al., 2024). The GO item and KEGG pathway (*p* < 0.05) were considered effective.

### 2.5 Molecular docking validation

The two-dimensional (2D) structure of the core ingredients of DWYG was obtained from the PubChem database (Kim et al., 2023). A three-dimensional (3D) structure was constructed using ChemBio3D and the structure of the small-molecule ligand was obtained after structural optimization. The 3D structure of the protein receptor was obtained from the PDB database (Berman et al., 2020). The water molecules and small-molecule ligands of the protein receptor were removed using PyMOL, and the protein receptor was hydrogenated to determine the active pocket using AutoDockTools (Morris et al., 2009). Molecular docking was performed to calculate the binding energy using AutoDock Vina. The molecular docking effect was relatively stable, and the binding activity is strong when the binding energy is < −5 kcal/mol. The specific molecular docking position was identified and visualized in 3D using PyMOL software.

### 2.6 *In vitro* and *in vivo* experiments

Twenty Sprague-Dawley (SD) rats, comprising an equal number of males and females, were used in this study. The rats were randomly and evenly divided into two groups: DWYG-G and blank control (BC-G). Rats in the BC-G group were fed 20 mL of 0.9% saline, and rats in the DWYG-G group were fed 20 mL of DWYG twice daily for 3 days. Prior to obtaining blood samples from the rats, a 12-h fast was implemented, while water was still permitted. Blood was collected via carotid artery catheterization 1 h after the rats were fed and kept awake. After allowing the blood to rest for 30 min, centrifugation was performed at 2,500 rpm for 15 min. Subsequently, the serum was transferred to a clean test tube and inactivated in a water bath at 56°C for 30 min. The serum was then divided into Eppendorf tubes with 10 mL per tube and stored at −20°C.

Approximately 10 mL of PMBCs was extracted from 10 HIV INRs and 10 HIV patients with normal immunologic reconstitution (NIR), and heparin anticoagulants were prepared. The PMBCs in the above two types of patients were isolated using the Ficoll method and added to medicine-containing serum and non-medicine-containing serum, respectively, and then cultured in a cell incubator. The groups were as follows. Group (1) comprised PBMC from NIR- and medicine-containing sera (NIR + M, n = 10). Group (2) comprised PBMC from NIR and non-medicinal serum (NIR + N, n = 10). Group (3) comprised PBMC from INR and medicine-containing serum (INR + M, n = 10). Group (4) comprised PBMC from INR and non-medicine sera (INR + N, n = 10).

RNA was extracted from the resuspended cells using TRIzol reagent. qRT-PCR was performed using the same amount of RNA and the One-Step PrimeScript RT-PCR Kit. The relative levels of TLR4 in different samples were calculated, and their trends were analyzed using the 2^−△△CT^ method using GAPDH as the reference gene. Flow cytometry was used to detect the content of major cytokines in the supernatant.

## 3 Results

### 3.1 Baseline characteristics of the study patients

As shown in Table 1, no differences in baseline characteristics were found among the 62 patients in this study.

**TABLE 1:** Baseline characteristics of the study patients.

Characteristics	Intensive group (n = 31)	Control group (n = 31)	*p*
Age (years)	53.10 (34.80–62.40)	44.10 (35.80–57.90)	0.5380
Male, n (%)	24 (77.42)	29 (93.55)	0.1466
Height (cm)	170.0 (160.0–173.0)	170.0 (167.0–175.0)	0.1483
Weight (kg)	62.0 (55.0–68.0)	62.0 (60.0–67.0)	0.4276
Baseline laboratory data			
CD4 count (cells/µL)	146 (118–171)	152 (103–202)	0.8969
CD8 count (cells/µL)	597 (428–763)	585 (432–803)	0.7124
CD4/CD8 ratio	0.230 (0.183–0.421)	0.235 (0.177–0.308)	0.9920

### 3.2 Change in the CD4:CD8 Rrtio in the Tto Ggoups

As shown in Table 2, the ratio of CD4^+^ to CD8^+^T cells in patients in both groups increased after 24 weeks of treatment. Compared with the baseline, the increase in the CD4:CD8 ratio in the trial group was significantly greater than that in the control group (*p* < 0.05).

**TABLE 2:** Change in the CD4:CD8 ratio in the two groups.

CD4/CD8 ratio [ M (P_25_, P_75_)]	Intensive group (n = 31)	Control group (n = 31)	*p*
Baseline mean	0.230 (0.183–0.421)	0.235 (0.177–0.308)	0.9920
Change in CD4/CD8 ratio at week 24 from baseline	0.023 (−0.011–0.064)^▲^	0.020 (0.001–0.042)^▲^	<0.0001

^▲^: Intra group comparison *p* < 0.05.

### 3.3 Flow cytometric analysis in the two groups

**FIGURE 1:**
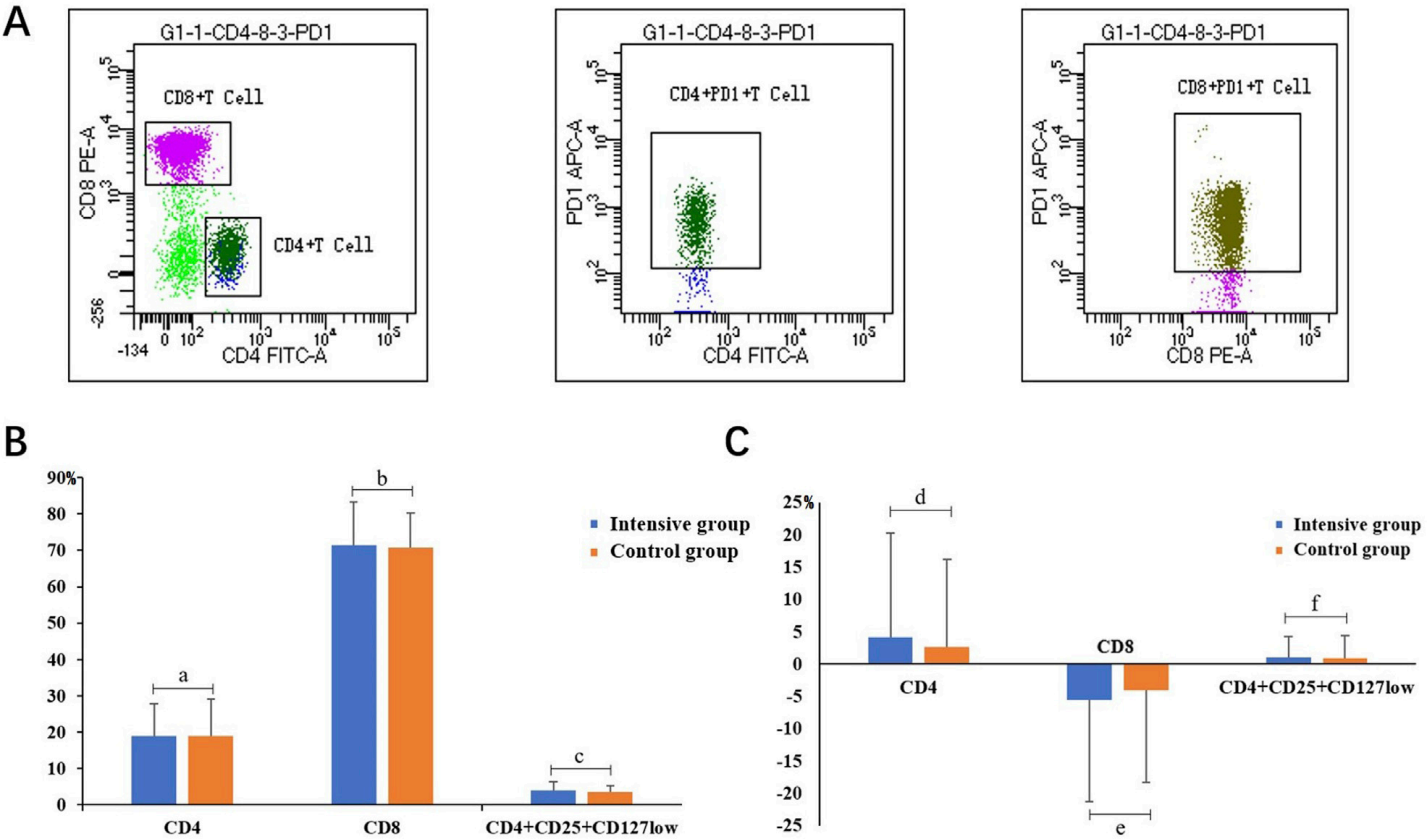
**(A)** Representative graph of CD4 and CD8 cell proportions measured by flow cytometry. **(B)** Comparison of the proportion of CD4, CD8, and CD4^+^CD25^+^CD127^low^ between the two groups at baseline. **(C)** Comparison of proportion difference of CD4, CD8, and CD4^+^CD25^+^CD127^low^ from baseline after 24 weeks; a *t* = −0.02, *p* = 0.9840; b *t* = 0.22, *p* = 0.8282; c *t* = 0.69, *p* = 0.4955; d *Z* = 0.1723, *p* < 0.0001; e *Z* = 0.7024, *p* < 0.0001; f *Z* = 0.0356, *p* < 0.0001.

As shown in Figure 1, the statistical analysis showed that after 24 weeks of treatment, the proportion of CD4^+^ and CD4^+^CD25^+^CD127^low^ in both groups showed an upward trend. Moreover, compared to the control group, the increase in the experimental group was more significant with respect to the proportion of CD4 (mean 4.057% vs 2.670%, *p* < 0.01) and CD4^+^CD25^+^CD127^low^ (mean 0.99% vs 0.867%, *p* < 0.01). The proportion of CD8 in the two groups showed a downward trend, and compared to the control group, the decrease in the experimental group was more significant (mean −5.54% vs −4.03%, *p* < 0.01).

### 3.4 Target prediction of potential compounds of DWYG against INR

**FIGURE 2:**
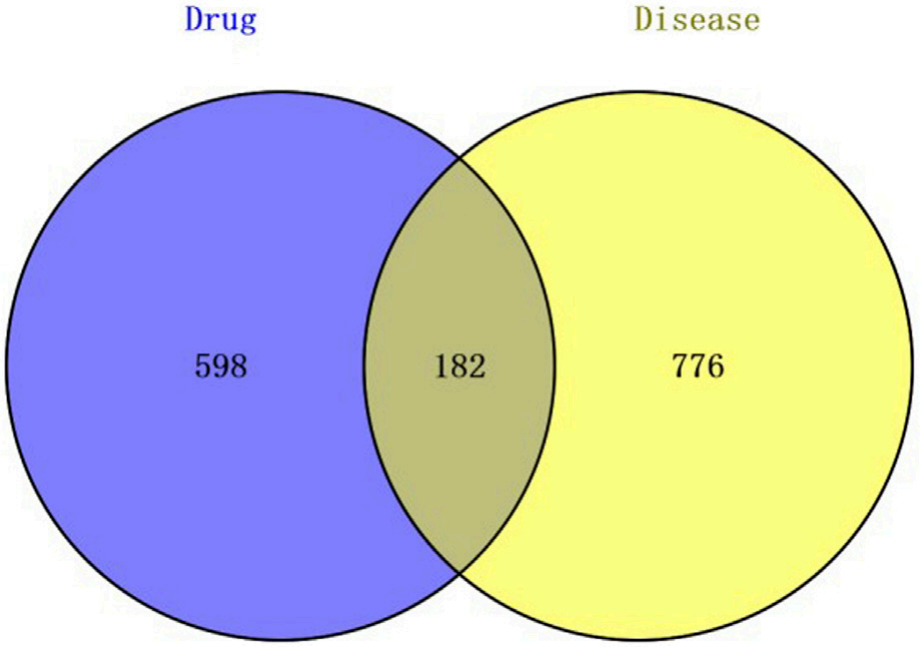
Venn diagram.

The compounds in the 80% methanol extract of DWYG were analyzed by UPLC-QTOF-MS/MS. A total of 210 chemical components were identified. The number of compounds from JH, SGC, YC, WWZ and SDH were 82, 56, 15, 43 and 4, respectively (Shi et al., 2023). Extraction-ion chromatograms for each compound are shown in Supplementary Figure S1, and chemical information for the 210 components in DWYG are shown in Supplementary Table S1. A total of 780 alternative compound targets were obtained by searching 210 chemical components using the Swiss Target Prediction and TCMSP databases. Furthermore, 958 INR-associated targets were retrieved from the GeneCard database. Additionally, 182 potential therapeutic targets of DWYG were identified using the Venn diagram, as depicted in Figure 2.

**FIGURE 3:**
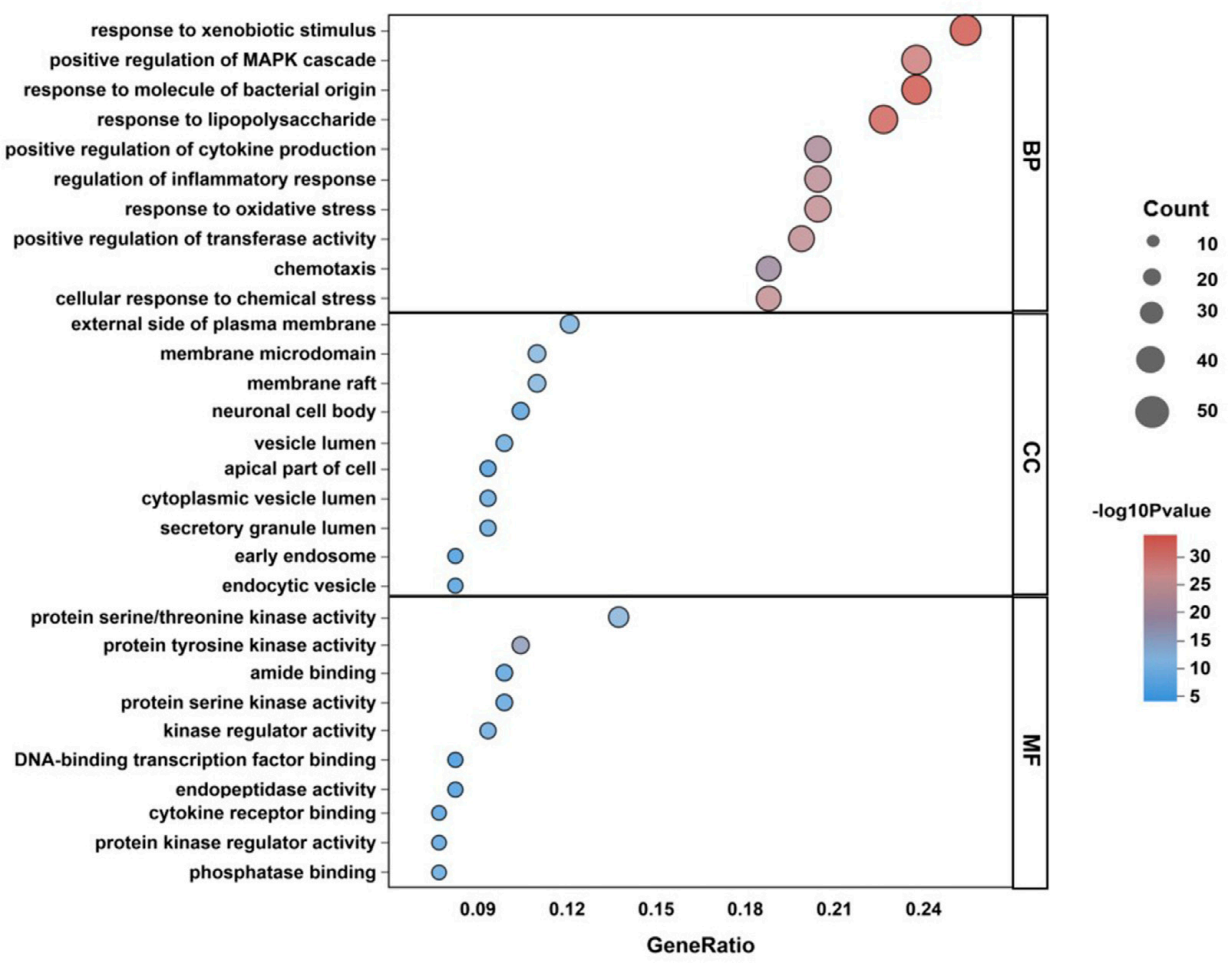
GO analysis.

GO enrichment analysis of 182 potential therapeutic targets was performed (Figure 3). The first ten items were plotted in terms of the horizontal gene ratio and vertical count. The biological process mainly involved response to xenobiotic stimulus, response to molecules of bacterial origin, and positive regulation of the MAPK cascade, while the cellular component mainly involved the external side of the plasma membrane, membrane raft, membrane microdomain, and molecular function (MF), which mainly involved protein serine/threonine kinase activity, protein tyrosine kinase activity, and amide binding.

**FIGURE 4:**
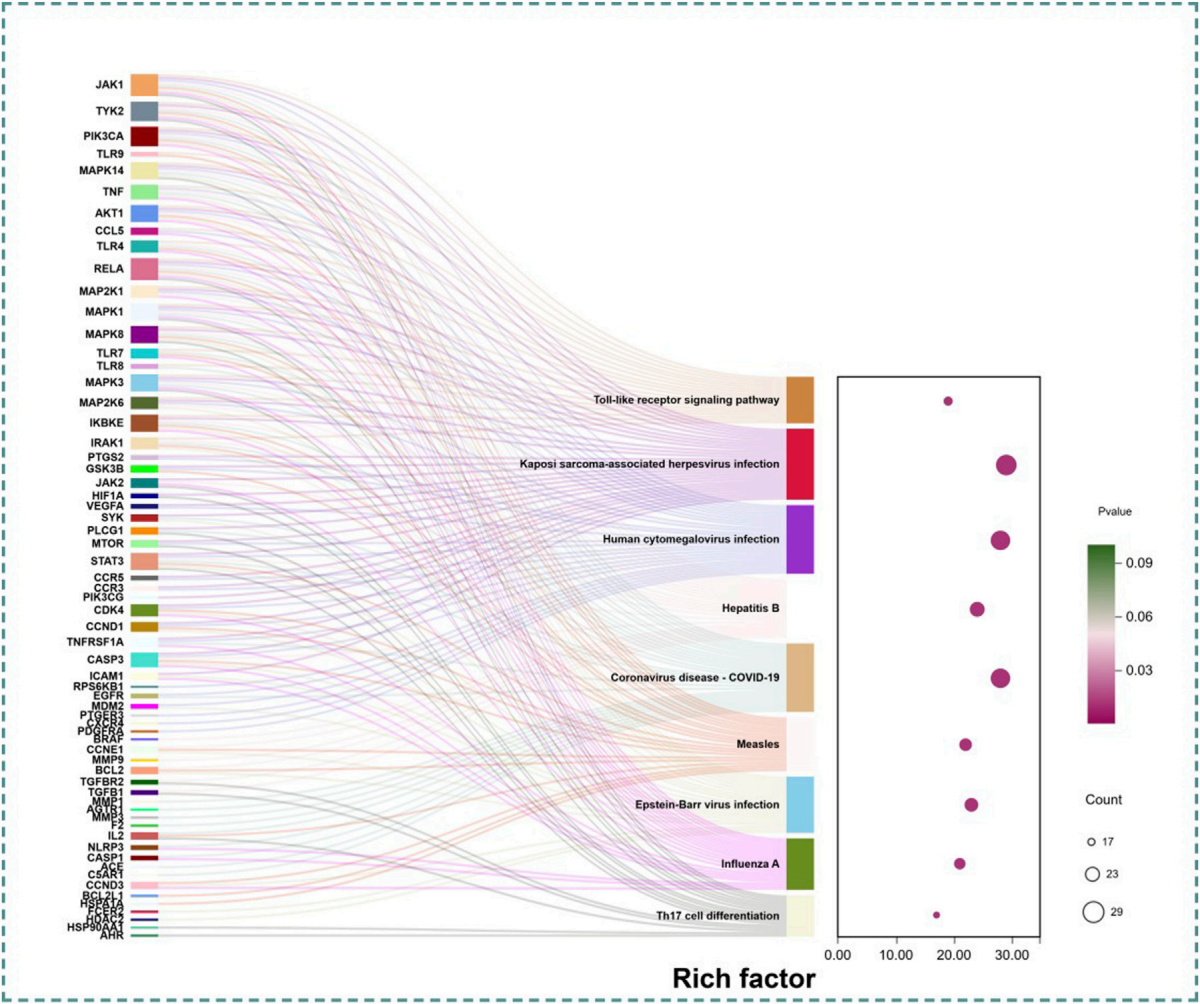
KEGG analysis.

As shown in Figure 4, KEGG pathway analysis showed that 182 potential therapeutic targets were involved in 173 signaling pathways. The first 30 pathways sorted by *p*-values were further analyzed. Because the main target disease of this study was INR, the signaling pathways related to other diseases, such as cardiovascular disease and cancer (specific types), were removed, and the signaling pathways of infectious disease (viral and immune system subcategory) were retained. Including the Toll-like receptor signaling pathway, Th17 cell differentiation, Kaposi sarcoma-associated herpesvirus infection, human cytomegalovirus infection, hepatitis B, coronavirus disease-COVID-19, measles, Epstein-Barr virus infection, and influenza A, i.e., total of nine pathways were retained.

**FIGURE 5:**
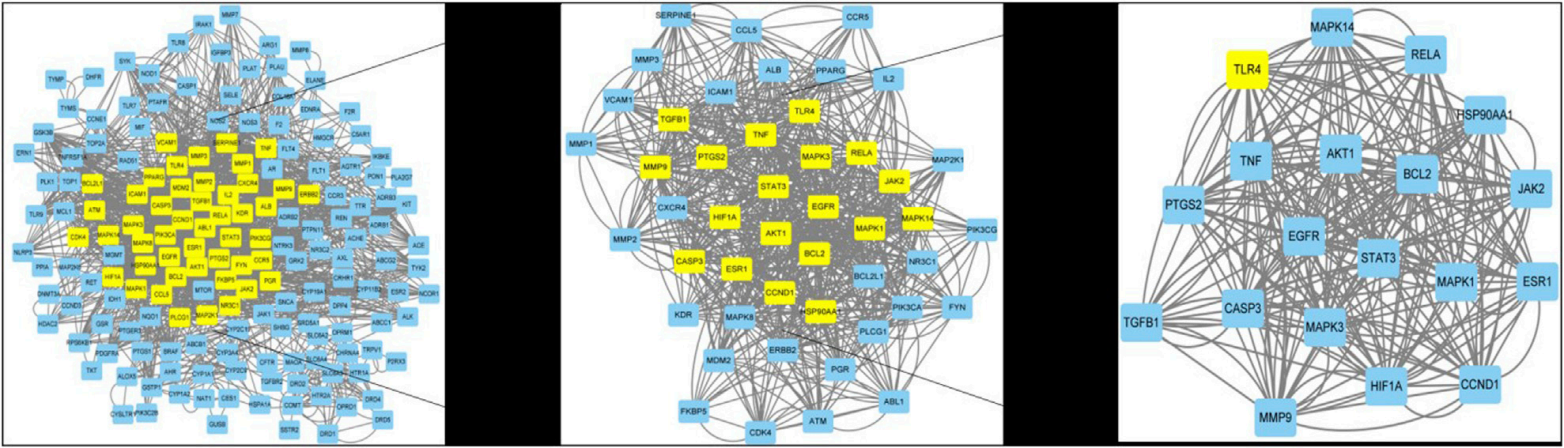
PPI results.

The 182 potential therapeutic targets were analyzed using STRING to further explore the interactions between the potential target proteins, which were screened to further explore the mechanism of action of the drug. The minimum required interaction score was set to high confidence (0.7) and network topology analysis was performed on all identified therapeutic targets to build an interaction network. The network consisted of 181 nodes and 1,091 edges with an average node degree of 12.1. Moreover, network centrality analysis was performed using CytoNCA and R programming languages, and 19 nodes and 264 edges were obtained, as shown in Figure 5. The central proteins of the PPI network included TLR4, TNF, STAT3, and JAK2, which may play important roles in DWYG’s promotion of immune reconstitution in DWYG.

### 3.5 Molecular docking analysis

**FIGURE 6:**
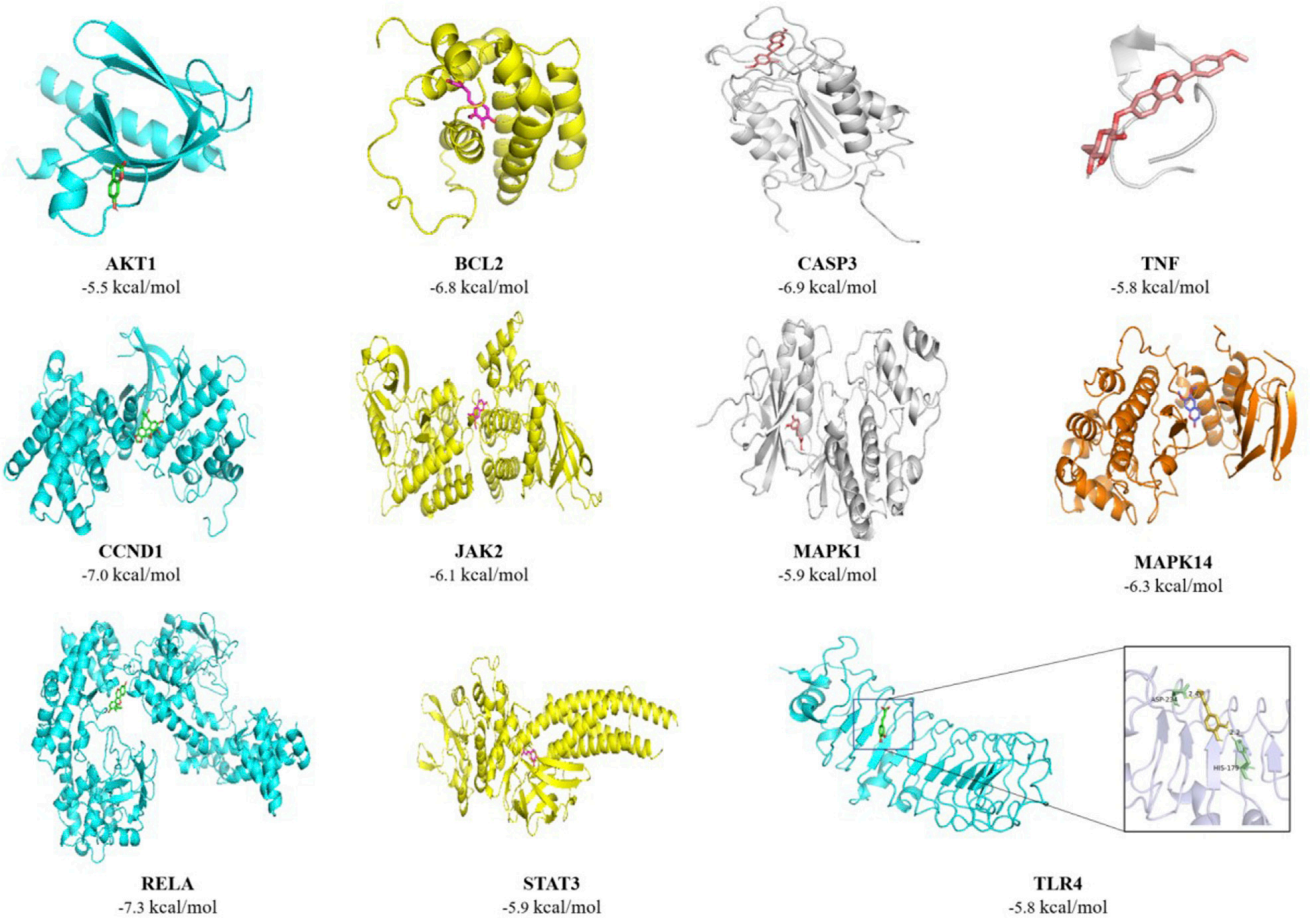
Molecular docking result.

As shown in Figure 6, combined with the GO and KEGG enrichment results, further molecular docking verification of TNF, MAPK14, CASP3, STAT3, MAPK3, TLR4, RELA, CCND1, JAK2, MAPK1, BCL2, and AKT1 was performed, and the results showed that the binding energy was < −5 kcal/mol.

### 3.6 Comparison of expression level of TLR4 in groups (1–4)

As shown in Table 3, the expression level of TLR4 was higher in Group (4) (INR + N) than in Group (2) (NIR + N); however, the difference was not statistically significant (*p* > 0.05). The expression level of TLR4 was lower in Group (3) (INR + M) than in Group (1) (NIR + M) (*p* > 0.05).

**TABLE 3:** RQ values of TLR4 in different groups (X ± SD).

	NIR + M	NIR + N	INR + M	INR + N	F	*p*
TLR4	0.6880 ± 0.6410	1.2697 ± 1.6492	1.7869 ± 1.5329	2.1094 ± 1.7657	1.440	0.252

^a^ RQ, value are calculated by2^−△△Ct^ method.

### 3.7 Comparison of major cytokine content in four groups

Table 4 shows significant differences in the levels of IL-2, IL-10, and TNF-α among the four groups. In particular, the expression level of IL-2 in Group (3) (INR + M) was lower than that in Group (4) (INR + N) (*p* = 0.012). Moreover, the expression level of IL-10 in Group (2) (NIR + N) was lower than that in Group (4) (INR + N) (*p* = 0.009). Additionally, the expression level of TNF-α in Group (1) (NIR + M) was lower than that in Group (3) (INR + M) (*p* = 0.029), and the expression level of TNF-α in Group (2) (NIR + N) was lower than that in Group (4) (INR + N) (*p* = 0.002).

**TABLE 4:** Analysis of main cytokines in different groups (X ± SD).

	NIR + M	NIR + N	INR + M	INR + N	F	*p*
IL-2	0.0762 ± 0.0844	0.1520 ± 0.1839	0.0937 ± 0.0504^a^	0.2752 ± 0.1414	5.208	0.009
IL-6	1.5912 ± 0.5410	1.4710 ± 0.4716	1.4048 ± 0.7514	1.3405 ± 0.5824	0.323	0.809
IL-10	0.5350 ± 0.6356	0.6111 ± 0.7422^b^	1.2769 ± 0.9841	2.1368 ± 1.4623	4.076	0.021
TNF-α	0.6636 ± 0.7436^c^	0.6085 ± 0.5762^b^	1.6560 ± 0.8264	1.9227 ± 0.8546	7.932	<0.001

^a^ INR + M VS INR + N, *p* < 0.05.

^b^ NIR + N VS INR + N.

^c^ NIR + M VS INR + M.

## 4 Discussion

CD4 is an important indicator for evaluating the immune function of patients with HIV. However, CD4 levels are easily affected by the internal environment and become unstable. Therefore, the CD4:CD8 ratio was observed at the same time. According to our previous clinical study, DWYG could effectively increase the proportion of CD45RA^+^ in INRs and stabilize thymic function. This clinical study and flow cytometry analysis further showed that the CD4 proportion and CD4/CD8 ratio in INRs were significantly increased after DWYG treatment, suggesting that DWYG can effectively restore immune function. When the number or function of CD4^+^CD25^+^CD127^Low^ cells is abnormal, autoimmune disorders can occur. This study found that DWYG treatment increased the number of CD4^+^CD25^+^CD127^Low^ cells.

DWYG consists of SDH, WWZ, YC, JH, and SGC, in which flavonoids, triterpenoids, and lignans are common components with proven efficacy against inflammation and liver protection. The main ingredients, such as caffeic acid, Ononin, Scoparone, and Licochalcone B, as analyzed by UPLC-QTOF-MS/MS, exert excellent anti-inflammatory effects by inducing leukocyte apoptosis and regulating inflammasome activation.

In this study, network pharmacology and molecular docking of DWYG were investigated, which showed that the mechanism by which DWYG regulates INR is characterized by multiple components, targets, and pathways. KEGG enrichment analysis showed that the pathways of DWYG regulating INR were mainly enriched in two types of subcategory of the immune system and viral infectious diseases. The immune system consists of the toll-like receptor signaling pathway and Th17 cell differentiation pathway. The toll-like receptor signaling pathway is composed of MYD88-dependent and MYD88-independent pathways. There were 19 enriched genes, including JAK1, TLR9, TNF, TLR4, and TLR7, which induced different signaling responses through different linkers. These signaling pathways include the regulation of inflammatory responses, cytokine release, and T-cell activation and proliferation to maintain immune balance. Th17 cell differentiation includes 17 enriched genes, such as IL-2, PLCG1, and STAT3. When the activation pathway of Th17 cell differentiation is abnormal, plasticity is affected, and the secretion of cytokines such as IL-17 and IL-10 is affected, thus leading to an excessive inflammatory response (Mills, 2023). Infectious diseases, such as viral infections, mainly include Kaposi sarcoma-associated herpesvirus and human cytomegalovirus, which have 29 and 28 genes clustered in the pathway, respectively, and are closely related to AIDS. In addition, Kaposi sarcoma and cytomegalovirus infections often occur in patients with compromised immune systems, and are common complications in AIDS patients with immunological reconstitution insufficiency.

The central proteins obtained from PPI enrichment analysis showed that the active ingredients of DWYG promoted immune reconstitution through multiple targets, such as TLR4, TNF, and STAT3. The expression of TLR4, one of the central proteins, was consistent with the KEGG enrichment results. DWYG can regulate the expression of many pro-inflammatory mediators such as Interleukin-6 (IL-6) and tumor necrosis factor-alpha (TNF-α), through TLR4 to suppress inflammatory responses and further regulate the immune status of patients. The central protein TNF is enriched in several major KEGG pathways, including Kaposi sarcoma-associated herpesvirus infection, human cytomegalovirus infection, and the toll-like receptor signaling pathway, and is a key cytokine that regulates the immune response and inflammation. DWYG can inhibit the expression of genes related to inflammation by simultaneously inhibiting the excessive or continuous activation of TNF and cell apoptosis to avoid the induction and amplification of inflammatory responses and hinder the recovery of immune function. In addition, the binding energies of the central protein targets (which were selected by combining the enrichment results of GO and KEGG) with the corresponding active ingredients were all < −5 kcal/mol, showing stable bonding characteristics, and TLR4 and TNF were the core targets of DWYG to promote immune reconstruction.

TLR4 was selected as the anchor point to further explore the mechanism of DWYG in the treatment of INR. The results showed that in the two groups of non-medicine serum samples, the level of TLR4 was lower in the NIR group, and the levels of the cytokines IL-2, IL-10, and TNF-α were lower than those in the INR group. In the two groups with INR, the expression of TLR4 was effectively downregulated after treatment with DWYG serum, and the levels of IL-2, IL-10, and TNF-α were significantly reduced. This indicates that DWYG can inhibit the overactivation of immune function and plays a significant role in the recovery or reconstruction of immune function in patients with AIDS by downregulating the expression of TLR4 in peripheral blood monocytes and related signaling pathways.

Further study on the role of TLR4 and TLRs in restoring immune function in INRs can provide a new and important theoretical basis for the research and development of Chinese medicine immune regulation drugs, and provide new methods and approaches for the clinical treatment of AIDS.

## 5 Conclusion

In summary, DWYG significantly increased the proportion of CD4^+^ T cells and CD4:CD8 ratio in INRs through multiple components, targets, and pathways. Moreover, the core targets were found to be TLR4 and TNF, and the core mechanism can be described as follows: DWYG downregulates the TLR4 level of INRs and the contents of related cytokines IL-2, IL-10, and TNF-α, inhibits the overactivation of immune function, and thereby promotes the reconstruction of immune function.

## Data Availability

The original contributions presented in the study are included in the article/Supplementary Material, further inquiries can be directed to the corresponding authors.

## References

[B1] Anzinger J. J. Butterfield T. R. Angelovich T. A. Crowe S. M. Palmer C. S. (2014). Monocytes as regulators of inflammation and HIV-related comorbidities during cART. J. Immunol. Res. 2014, 569819. 10.1155/2014/569819 25025081 PMC4082935

[B2] Berman H. M. Westbrook J. Feng Z. Gilliland G. Bhat T. N. Weissig H. (2000). The protein data bank. Nucleic Acids Res. 28 (1), 235–242. 10.1093/nar/28.1.235 10592235 PMC102472

[B3] Brubaker S. W. Bonham K. S. Zanoni I. Kagan J. C. (2015). Innate immune pattern recognition: a cell biological perspective. Annu. Rev. Immunol. 33, 257–290. 10.1146/annurev-immunol-032414-112240 25581309 PMC5146691

[B4] Fitzgerald K. A. Kagan J. C. (2020). Toll-like receptors and the control of immunity. Cell 180 (6), 1044–1066. 10.1016/j.cell.2020.02.041 32164908 PMC9358771

[B5] Gfeller D. Grosdidier A. Wirth M. Daina A. Michielin O. Zoete V. (2014). SwissTargetPrediction: a web server for target prediction of bioactive small molecules. Nucleic Acids Res. 42 (Web Server issue), W32–W38. 10.1093/nar/gku293 24792161 PMC4086140

[B6] Ji S. X. Zhang H. Y. Wan T. J. Wen L. Feng Q. S. (2022). Role of traditional Chinese medicine in incomplete immune reconstitution of HIV/AIDS: a review. Tradit. Med. Res. 7 (6), 50. 10.53388/TMR20220214001

[B7] Kanehisa M. Goto S. (2000). KEGG: kyoto encyclopedia of genes and genomes. Nucleic Acids Res. 28 (1), 27–30. 10.1093/nar/28.1.27 10592173 PMC102409

[B8] Kim S. Chen J. Cheng T. Gindulyte A. He J. He S. (2023). PubChem 2023 update. Nucleic Acids Res. 51 (D1), D1373–D1380. 10.1093/nar/gkac956 36305812 PMC9825602

[B9] Lei E. Jin S. Ni W. Feng M. Luo Y. Ruan L. (2022). Analysis of the influencing factors of immunological nonresponders in wuhan, China. Can. J. Infect. Dis. Med. Microbiol. 2022, 5638396. 10.1155/2022/5638396 35979516 PMC9377976

[B10] Lei E. Z. Ruan L. G. Chen Y. Ni W. Yu X. L. Hong K. (2022). Effect of Diwu Liver-Nourishing Capsule combine with HAART on HIV/AIDS patients with poor immune reconstitution. Chin. J. AIDS and STD. 28(10):1127–1131. 10.13419/j.cnki.aids.2022.10.03

[B11] Mills K. H. G. (2023). IL-17 and IL-17-producing cells in protection versus pathology. Nat. Rev. Immunol. 23 (1), 38–54. 10.1038/s41577-022-00746-9 35790881 PMC9255545

[B12] Morris G. M. Huey R. Lindstrom W. Sanner M. F. Belew R. K. Goodsell D. S. (2009). AutoDock4 and AutoDockTools4: automated docking with selective receptor flexibility. J. Comput. Chem. 30 (16), 2785–2791. 10.1002/jcc.21256 19399780 PMC2760638

[B13] Rb-Silva R. Goios A. Kelly C. Teixeira P. João C. Horta A. (2019). Definition of immunological nonresponse to antiretroviral therapy: a systematic review. J. Acquir Immune Defic. Syndr. 82 (5), 452–461. 10.1097/QAI.0000000000002157 31592836

[B14] Roul H. Mary-Krause M. Ghosn J. Delaugerre C. Pialoux G. Cuzin L. (2018). CD4^+^cell count recovery after combined antiretroviral therapy in the modern combined antiretroviral therapy era. AlDS 32 (17), 2605–2614. 10.1097/QAD.0000000000002010 30289817

[B15] Ru J. Li P. Wang J. Zhou W. Li B. Huang C. (2014). TCMSP: a database of systems pharmacology for drug discovery from herbal medicines. J. Cheminform 6, 13. 10.1186/1758-2946-6-13 24735618 PMC4001360

[B25] Shi Q. He J. Chen G. Xu J. Zeng Z. Zhao X. (2023). The chemical composition of Diwu YangGan capsule and its potential inhibitory roles on hepatocellular carcinoma by microarray-based transcriptomics. J. Tradit. Complement. Med. 14 (4), 381–390. 10.1016/j.jtcme.2023.12.002 39035694 PMC11259662

[B16] Safran M. Dalah I. Alexander J. Rosen N. Iny Stein T. Shmoish M. (2010). GeneCards Version 3: the human gene integrator. Database (Oxford) 2010, baq020. 10.1093/database/baq020 20689021 PMC2938269

[B17] Sokoya T. Steel H. C. Nieuwoudt M. Rossouw T. M. (2017). HIV as a cause of immune activation and immunosenescence. Mediat. Inflamm. 2017, 6825493. 10.1155/2017/6825493 PMC5676471 29209103

[B18] Szklarczyk D. Kirsch R. Koutrouli M. Nastou K. Mehryary F. Hachilif R. (2023). The STRING database in 2023: protein-protein association networks and functional enrichment analyses for any sequenced genome of interest. Nucleic Acids Res. 51 (D1), D638–D646. 10.1093/nar/gkac1000 36370105 PMC9825434

[B19] Tang Y. Li M. Wang J. Pan Y. Wu F. X. (2015). CytoNCA: a cytoscape plugin for centrality analysis and evaluation of protein interaction networks. Biosystems 127, 67–72. 10.1016/j.biosystems.2014.11.005 25451770

[B20] Xu S. Hu E. Cai Y. Xie Z. Luo X. Zhan L. (2024). Using clusterProfiler to characterize multiomics data. Nat. Protoc. 10.1038/s41596-024-01020-z 39019974

[B21] Xu W. Xiao M. Li J. Chen Y. Sun Q. Li H. (2020). Hepatoprotective effects of Di Wu Yang Gan: a medicinal food against CCl4-induced hepatotoxicity *in vivo* and *in vitro* . Food Chem. 327, 127093. 10.1016/j.foodchem.2020.127093 32470802

[B22] Yang X. Su B. Zhang X. Liu Y. Wu H. Zhang T. (2020). Incomplete immune reconstitution in HIV/AIDS patients on antiretroviral therapy: challenges of immunological non-responders. J. Leukoc. Biol. 107 (4), 597–612. 10.1002/JLB.4MR1019-189R 31965635 PMC7187275

[B23] Zhang C. Xia S. Yan M. Luo F. Zhang B. Zou W. (2023). Bioinformatics and network pharmacology analysis of DWYG capsule for improving liver regeneration: identification of active compounds and mechanisms. Nat. Prod. Res. 38, 3329–3335. 10.1080/14786419.2023.2246630 37574795

[B24] Zhang Q. Yu X. Wu T. Shang H. Jiang Y. (2020). Immunological and virological responses in older HIV-infected adults receiving antiretroviral therapy: an evidence-based meta-analysis. J. Acquir lmmune Defic. Syndr. 83 (4), 323–333. 10.1097/QAI.0000000000002266 31913990

