# Supplementary material for: Investigation on improving immunologic reconstitution insufficiency using DiwuYanggan capsules in AIDS patients
Source: Front Pharmacol. 2024 Nov 6;15:1485719. doi: 10.3389/fphar.2024.1485719 (PMC11576195; doi:10.3389/fphar.2024.1485719)
Supplement: Supplementary file 1 [file Table1.docx]

Supplementary Material

# Supplementary Table

## Supplementary Table 1

|  | Name | Molecular formula | [M+H^+^] | The measured values | ppm | Retention time | Secondary fragment |
| --- | --- | --- | --- | --- | --- | --- | --- |
| 1^a^ | 1,7-bis(4-hydroxyphenyl)heptane-3,5-diol | C_19_H_25_O_4_ | 317.1753 | 317.1726 | -8.51 | 18.00 | 93.0324, 107.0490, 209.1171, 195.1076, 151.0764, 165.0884 |
| 2^a^ | 4-(3,5-dihydroxy-7-phenylheptyl)benzene-1,2-diol | C_19_H_25_O_4_ | 317.1753 | 317.1726 | -8.51 | 20.02 | 137.0573, 179.1048, 181.0849, 135.0808, 105.0701, 91.0533, 225.1082 |
| 3^a^ | 4-(3,5-dihydroxy-7-(4-hydroxyphenyl)heptyl)benzene-1,2-diol | C_19_H_25_O_5_ | 333.1702 | 333.1680 | -6.60 | 21.19 | 137.0597, 165.0936, 181.0849, 151.0764 |
| 4^a^ | 5-(3,5-dihydroxy-7-phenylheptyl)benzene-1,2,3-triol | C_19_H_25_O_5_ | 333.1702 | 333.1680 | -6.60 | 18.83 | 125.0239, 179.1048, 149.0933, 105.0680, 241.1065, 77.0395 |
| 5^a^ | 1-(4-hydroxy-3-methoxyphenyl)-7-(4-hydroxyphenyl)heptane-3,5-diol | C_21_H_27_O_4_ | 343.1909 | 343.1929 | 5.83 | 27.00 | 77.0377, 123.0456, 151.0739, 191.1064, 147.0786, 195.1047, 103.0534 |
| 6^a^ | 1-(3,4-dimethoxyphenyl)-7-(4-hydroxy-3,5-dimethoxyphenyl)heptane-3,5-diol | C_23_H_33_O_7_ | 421.2226 | 421.2199 | -6.41 | 17.19 | 167.0716, 209.1171, 195.1047, 165.0936, 137.0597 |
| 7^a^ | 5-(3,5-dihydroxy-7-(4-hydroxy-2,3,5-trimethoxyphenyl)heptyl)benzene-1,2,3,4-tetraol | C_22_H_31_O_10_ | 455.1917 | 455.1922 | 1.10 | 31.86 | 313.1646, 155.0335, 299.1503, 211.0986, 183.0649, 169.0534 |
| 8^a^ | 1-(3,4-dimethoxyphenyl)-7-(2,3,4,5-tetramethoxyphenyl)heptane-3,5-diol | C_25_H_37_O_8_ | 465.2488 | 465.2495 | 1.50 | 37.05 | 253.1440, 209.1201, 165.0936, 327.1812, 137.0597 |
| 9^a^ | (E)-1,7-diphenylhept-1-ene-3,5-diol | C_19_H_23_O_2_ | 283.1698 | 283.1679 | -6.71 | 19.86 | 77.0377, 191.1064, 91.0533, 177.0539, 105.0701, 147.0786, 135.0832, 149.0933, 133.0619, 103.0534, 179.1048, 265.1614 |
| 10^a^ | (E)-1,7-bis(4-hydroxyphenyl)hept-1-ene-3,5-diol | C_19_H_23_O_4_ | 315.1596 | 315.1568 | -8.88 | 22.24 | 193.0862, 163.0725, 149.0558, 165.0910, 119.0470, 195.0990 |
| 11^a^ | (E)-4-(3,5-dihydroxy-7-phenylhept-6-en-1-yl)benzene-1,2-diol | C_19_H_23_O_4_ | 315.1596 | 315.1568 | -8.88 | 22.93 | 177.0920, 147.0786, 181.0849, 133.0643, 103.0514, 123.0456, 161.0924 |
| 12^a^ | (E)-7-(3,4-dimethoxyphenyl)-1-(4-hydroxyphenyl)hept-1-ene-3,5-diol | C_21_H_27_O_5_ | 359.1858 | 359.1834 | -6.68 | 21.00 | 195.1047, 163.0751, 149.0583, 119.0470, 177.0920, 147.0811 |
| 13^a^ | (E)-5-methoxy-1-(4-methoxyphenyl)-7-phenylhept-1-en-3-ol | C_21_H_27_O_3_ | 327.1960 | 327.1963 | 0.92 | 21.57 | 105.0701, 149.0958, 177.0920, 163.1117, 133.0643, 161.0950 |
| 14^a^ | (E)-5-methoxy-7-(4-methoxyphenyl)-1-phenylhept-1-en-3-ol | C_21_H_27_O_3_ | 327.1960 | 327.1972 | 3.67 | 25.97 | 191.1064, 135.0784, 179.1048, 149.0836, 193.1260, 103.0534, 121.0628 |
| 15^a^ | (E)-4-(5-hydroxy-3-methoxy-7-phenylhept-6-en-1-yl)-2-methoxyphenol | C_21_H_27_O_4_ | 343.1909 | 343.1929 | 5.83 | 26.59 | 123.0456, 151.0714, 191.1092, 147.0811, 195.0990, 103.0534, 77.0377 |
| 16^a^ | (E)-5-methoxy-7-(4-methoxyphenyl)-1-(3,4,5-trimethoxyphenyl)hept-1-en-3-ol | C_24_H_33_O_6_ | 417.2277 | 417.2269 | -1.92 | 26.69 | 107.0490, 121.0651, 135.0832, 281.1340, 179.1048, 237.1161, 193.1203, 293.1738, 265.1414, 207.1361, 167.0716 |
| 17^a^ | (E)-4-(7-(3,4-dimethoxyphenyl)-5-hydroxy-3-methoxyhept-6-en-1-yl)-2,3,6-trimethoxyphenol | C_25_H_35_O_8_ | 463.2332 | 463.2365 | 7.12 | 37.08 | 279.1576, 197.0821, 251.1261, 163.0777, 137.0597, 325.1706, 445.2206, 249.1517, 191.1035, 177.0893, 309.1756 |
| 18^a^ | (E)-4,4'-(3-hydroxyhept-1-ene-1,7-diyl)diphenol | C_19_H_23_O_3_ | 299.1647 | 299.1625 | -7.35 | 23.48 | 191.1064, 177.0866, 135.0808, 149.1008, 149.0583, 119.0470, 179.1048, 189.1255, 175.1123, 147.0811, 133.0643 |
| 19^a^ | (E)-4-(3-hydroxy-7-phenylhept-1-en-1-yl)benzene-1,2-diol | C_19_H_23_O_3_ | 299.1647 | 299.1625 | -7.35 | 20.02 | 77.0395, 91.0533, 105.0701, 119.0850, 179.0665, 133.0997, 135.0427, 163.1091, 191.1064, 163.0777, 149.0608, 147.1208 |
| 20^a^ | (E)-1,7-bis(4-methoxyphenyl)hept-1-en-3-ol | C_21_H_27_O_3_ | 327.1960 | 327.1963 | 0.92 | 24.98 | 135.0832, 191.1035, 177.0920, 133.0619, 121.0628 |
| 21^a^ | (E)-4-(3-hydroxy-7-(4-hydroxyphenyl)hept-1-en-1-yl)-2-methoxyphenol | C_20_H_25_O_4_ | 329.1753 | 329.1759 | 1.82 | 26.69 | 93.0343, 107.0490, 207.1007, 135.0832, 193.0890, 149.0958, 179.0720, 149.0583, 179.1048, 163.1117, 177.0893 |
| 22^a^ | (E)-4-(5-hydroxy-7-(4-hydroxyphenyl)hept-6-en-1-yl)-2-methoxyphenol | C_20_H_25_O_4_ | 329.1753 | 329.1722 | -9.42 | 30.03 | 177.0866, 151.0739, 123.0434, 163.0777, 165.0910, 179.1076, 119.0493, 209.1171, 147.0786, 161.0950, 175.1123 |
| 23^a^ | 1-(4-hydroxy-3-methoxyphenyl)-7-(4-hydroxyphenyl)heptane-3,5-dione | C_20_H_23_O_5_ | 343.1545 | 343.1550 | 1.46 | 14.95 | 137.0597, 161.0587, 151.0739, 191.0696, 147.0414, 119.0493 |
| 24^a^ | 1-(4-hydroxy-3-methoxyphenyl)-7-(4-hydroxyphenyl)heptane-3,5-dione | C_20_H_23_O_5_ | 343.1545 | 343.1512 | -9.62 | 15.79 | 137.0573, 205.0824, 191.0696, 163.0804, 149.0583, 121.0651 |
| 25^a^ | (E)-1-(3,4-dimethoxyphenyl)-5-hydroxy-7-(3,4,5-trimethoxyphenyl)hept-1-en-3-one | C_24_H_31_O_7_ | 431.2070 | 431.2112 | 9.74 | 34.81 | 181.0905, 249.1097, 195.1076, 235.0942, 225.1082, 163.080, 267.1211 |
| 26^a^ | (E)-1,7-bis(4-hydroxyphenyl)hept-6-en-3-one | C_19_H_21_O_3_ | 297.1491 | 297.1484 | -2.36 | 25.81 | 133.0643, 163.0725, 149.0608, 147.0786 |
| 27^a^ | (E)-1-(3,4-dimethoxyphenyl)-7-(3,4,5-trimethoxyphenyl)hept-6-en-3-one | C_24_H_31_O_6_ | 415.2121 | 415.2108 | -3.13 | 20.33 | 193.0862, 207.1037, 263.1325, 137.0573, 277.1507 |
| 28^a^ | (E)-7-(3,4-dimethoxyphenyl)-1-(3,4,5-trimethoxyphenyl)hept-6-en-3-one | C_24_H_31_O_6_ | 415.2121 | 415.2105 | -3.85 | 24.02 | 177.0893, 237.1130, 223.0924, 195.1019, 167.0716, 163.0777 |
| 29^a^ | (E)-7-(4-methoxyphenyl)-1-(2,3,4,5-tetramethoxyphenyl)hept-6-en-3-one | C_24_H_31_O_6_ | 415.2121 | 415.2105 | -3.85 | 21.57 | 133.0643, 161.0950, 281.1375, 189.0889, 217.1234 |
| 30^a^ | (E)-7-(4-methoxyphenyl)-1-(2,3,4,5-tetramethoxyphenyl)hept-6-en-3-one | C_24_H_31_O_6_ | 415.2121 | 415.2105 | -3.85 | 22.74 | 307.1494, 133.0619, 281.1409, 147.0786, 161.0976, 253.1049, 225.1143, 203.1060, 197.0850, 167.0716 |
| 31^a^ | (E)-1-(3,4-dihydroxyphenyl)-5-(4-hydroxyphenyl)pent-1-en-3-one | C_17_H_17_O_4_ | 285.1127 | 285.1100 | -9.47 | 19.86 | 177.0539, 121.0651, 135.0427, 149.0583 |
| 32^a^ | (E)-7-hydroxy-1,7-bis(4-hydroxyphenyl)hept-1-ene-3,5-dione | C_19_H_19_O_5_ | 327.1232 | 327.1222 | -3.06 | 17.12 | 103.0513, 145.0679, 137.0579 |
| 33^a^ | (E)-7-(3,4-dihydroxyphenyl)-7-hydroxy-1-phenylhept-1-ene-3,5-dione | C_19_H_19_O_5_ | 327.1232 | 327.1222 | -3.06 | 17.97 | 77.0389, 103.0513, 131.0473, 145.0679, 153.0546, 187.0773 |
| 34^a^ | (E)-7-(3,4-dimethoxyphenyl)-7-hydroxy-1-(4-hydroxyphenyl)hept-1-ene-3,5-dione | C_21_H_23_O_6_ | 371.1495 | 371.1530 | 9.43 | 19.29 | 119.0493, 189.0580, 137.0597 |
| 35^a^ | (E)-1-(3,4-dimethoxyphenyl)-7-hydroxy-7-(4-hydroxy-3-methoxyphenyl)hept-1-ene-3,5-dione | C_22_H_25_O_7_ | 401.1600 | 401.1582 | -4.49 | 9.31 | 163.0751, 237.0752, 195.0676, 205.0824, 167.0743, 153.0549, 123.0434 |
| 36^a^ | (E)-7-hydroxy-1-(4-hydroxy-3,5-dimethoxyphenyl)-7-(3,4,5-trimethoxyphenyl)hept-1-ene-3,5-dione | C_24_H_28_O_9_ | 461.1812 | 461.1844 | 6.94 | 35.03 | 153.0524, 207.0683, 253.1049, 239.0961, 249.0710, 263.0960, 167.0716 |
| 37^a^ | (E)-1-(3,4-dihydroxy-5-methoxyphenyl)-7-hydroxy-7-(2,3,4,5-tetramethoxyphenyl)hept-1-ene-3,5-dione | C_24_H_29_O_10_ | 477.1761 | 477.1720 | -8.59 | 34.81 | 337.1131, 193.0463, 235.0597, 227.0899 |
| 38^a^ | (E)-1,7-bis(4-hydroxyphenyl)hept-1-ene-3,5-dione | C_19_H_19_O_4_ | 311.1283 | 311.1300 | 5.46 | 13.90 | 93.0324, 217.0903, 107.0490, 189.0608, 163.0751, 147.0439, 119.0515 |
| 39^a^ | (E)-1,7-bis(4-hydroxyphenyl)hept-1-ene-3,5-dione | C_19_H_19_O_4_ | 311.1283 | 311.1264 | -6.11 | 29.22 | 93.0363, 189.0580, 149.0583, 161.0587, 147.0464, 191.0668, 119.0470 |
| 40^a^ | (E)-7-(3,4-dimethoxyphenyl)-1-phenylhept-1-ene-3,5-dione | C_21_H_23_O_4_ | 339.1596 | 339.1599 | -0.88 | 34.50 | 137.0597, 201.1044, 151.0789, 165.0910, 173.0521, 145.0653, 131.0488, 103.0534, 261.1147, 77.0377 |
| 41^a^ | (E)-7-(4-hydroxy-3-methoxyphenyl)-1-(4-hydroxyphenyl)hept-1-ene-3,5-dione | C_20_H_21_O_5_ | 341.1389 | 341.1375 | -4.10 | 13.71 | 137.0597, 203.0681, 189.0580, 161.0612, 193.0833, 119.0470, 93.0343, 247.0951 |
| 42^a^ | (E)-7-(4-hydroxy-3-methoxyphenyl)-1-(4-hydroxyphenyl)hept-1-ene-3,5-dione | C_20_H_21_O_5_ | 341.1389 | 341.1380 | -2.64 | 19.08 | 189.0523, 179.0692, 161.0587, 193.0833, 147.0439, 119.0493, 221.0795 |
| 43^a^ | (E)-1-(4-hydroxy-3-methoxyphenyl)-7-(4-hydroxyphenyl)hept-1-ene-3,5-dione | C_20_H_21_O_5_ | 341.1389 | 341.1375 | -4.10 | 14.24 | 93.0343, 121.0628, 149.0608, 191.0696, 177.0539, 123.0456, 217.0842 |
| 44^a^ | (E)-1-(3,4-dimethoxyphenyl)-7-(4-hydroxy-3-methoxyphenyl)hept-1-ene-3,5-dione | C_22_H_25_O_6_ | 385.1651 | 385.1667 | 4.15 | 21.25 | 137.0597, 179.0747, 205.0824, 193.0833 |
| 45^a^ | (E)-1-(3,4-dihydroxy-5-methoxyphenyl)-7-(4-hydroxy-3-methoxyphenyl)hept-1-ene-3,5-dione | C_21_H_23_O_7_ | 387.1444 | 387.1424 | -5.17 | 34.91 | 123.0456, 263.0927, 249.0774, 151.0764, 179.0720, 193.0492, 165.0568, 139.0386 |
| 46^a^ | (E)-7-(4-methoxyphenyl)-1-(3,4,5-trimethoxyphenyl)hept-1-ene-3,5-dione | C_23_H_27_O_6_ | 399.1808 | 399.1810 | 0.50 | 32.20 | 277.0996, 163.0777, 235.1005, 177.0920, 221.0795, 205.0853, 231.0981 |
| 47^a^ | (E)-1-(3,4-dimethoxyphenyl)-7-(4-hydroxy-3,5-dimethoxyphenyl)hept-1-ene-3,5-dione | C_23_H_27_O_7_ | 415.1757 | 415.1771 | 3.37 | 23.40 | 153.0549, 261.1180, 247.0951, 181.0877, 233.0809, 209.0816, 191.0724, 163.0751 |
| 48^a^ | (E)-7-(4-hydroxy-2,3,5-trimethoxyphenyl)-1-(2,3,4,5-tetrahydroxyphenyl)hept-1-ene-3,5-dione | C_22_H_25_O_10_ | 449.1448 | 449.1480 | 7.12 | 21.19 | 239.0898, 167.0320, 141.0171 |
| 49^a^ | (E)-1-(4-hydroxy-2,3,5-trimethoxyphenyl)-7-(2,3,4,5-tetramethoxyphenyl)hept-1-ene-3,5-dione | C_26_H_33_O_10_ | 505.2074 | 505.2065 | -1.78 | 34.81 | 197.0850, 209.0816 |
| 50^a^ | (4E,6E)-1,7-bis(4-hydroxyphenyl)hepta-4,6-dien-3-one | C_19_H_19_O_3_ | 295.1334 | 295.1341 | 2.37 | 18.52 | 119.0493, 145.0653, 149.0608, 121.0651, 107.0490187.0766 |
| 51^a^ | (4E,6E)-1-(3,4-dimethoxyphenyl)-7-phenylhepta-4,6-dien-3-one | C_21_H_23_O_3_ | 323.1647 | 323.1666 | 5.88 | 24.14 | 77.0377, 103.0514, 129.0697, 193.0890, 157.0659, 171.0817, 151.0789, 185.1001 |
| 52^a^ | (4E,6E)-1-(4-hydroxy-3-methoxyphenyl)-7-(4-hydroxyphenyl)hepta-4,6-dien-3-one | C_20_H_21_O_4_ | 325.1440 | 325.1451 | 3.38 | 19.52 | 145.0653, 151.0739, 137.0597, 187.0794, 123.0434, 201.0870, 93.0324 |
| 53^a^ | (4E,6E)-7-(4-hydroxy-3-methoxyphenyl)-1-(4-hydroxyphenyl)hepta-4,6-dien-3-one | C_20_H_21_O_4_ | 325.1440 | 325.1451 | 3.38 | 16.76 | 201.0928, 149.0583, 175.0716, 121.0628, 217.0873, 93.0343, 231.1043 |
| 54^a^ | (4E,6E)-1,7-bis(4-hydroxy-3-methoxyphenyl)hepta-4,6-dien-3-one | C_21_H_23_O_5_ | 355.1545 | 355.1536 | -2.53 | 31.24 | 123.0456, 231.0981, 149.0608, 179.0720, 151.0764, 203.0681 |
| 55^a^ | (4E,6E)-1-(4-hydroxy-3,5-dimethoxyphenyl)-7-(4-hydroxy-3-methoxyphenyl)hepta-4,6-dien-3-one | C_22_H_25_O_6_ | 385.1651 | 385.1627 | -6.23 | 12.88 | 261.1147, 203.0740, 235.0973, 209.0786, 217.0842, 167.0716 |
| 56^a^ | (4E,6E)-1-(4-methoxyphenyl)-7-(2,3,4,5-tetramethoxyphenyl)hepta-4,6-dien-3-one | C_24_H_29_O_6_ | 413.1964 | 413.1947 | -4.11 | 22.09 | 215.1048, 249.1162, 163.0725, 135.0832, 291.1275 |
| 57^a^ | (4E,6E)-1,7-bis(3,4,5-trimethoxyphenyl)hepta-4,6-dien-3-one | C_25_H_31_O_7_ | 443.2070 | 443.2046 | -5.42 | 32.79 | 167.0716, 193.0833, 219.1001, 181.0849 |
| 58^a^ | (1E,4E)-1-(4-hydroxy-3-methoxyphenyl)-5-(4-hydroxyphenyl)penta-1,4-dien-3-one | C_18_H_17_O_4_ | 297.1127 | 297.1132 | 1.68 | 15.26 | 149.0608, 177.0539, 123.0411, 173.0521 |
| 59^a^ | (1E,4E)-1-(3,4-dihydroxyphenyl)-5-(4-methoxyphenyl)penta-1,4-dien-3-one | C_18_H_17_O_4_ | 297.1127 | 297.1132 | 1.68 | 22.19 | 189.0523, 133.0666, 163.0385, 161.0587, 187.0710 |
| 60^a^ | (1E,4E)-1-(3,4-dimethoxyphenyl)-5-(4-hydroxyphenyl)penta-1,4-dien-3-one | C_19_H_19_O_4_ | 311.1283 | 311.1264 | -6.11 | 20.64 | 137.0597, 191.0668, 119.0493, 93.0324 |
| 61^a^ | (E)-(E)-4-(4-hydroxy-3-methoxyphenyl)-2-oxobut-3-en-1-yl 3-(4-hydroxy-3-methoxyphenyl)acrylate | C_21_H_21_O_7_ | 385.1287 | 385.1278 | -2.34 | 24.88 | 193.0520, 177.0539, 207.0654 |
| 62^a^ | (E)-(E)-4-(4-methoxyphenyl)-2-oxobut-3-en-1-yl 3-(2,3,4,5-tetramethoxyphenyl)acrylate | C_24_H_27_O_8_ | 443.1706 | 443.1744 | 8.57 | 21.57 | 133.0643, 309.1000, 161.0612, 281.1031, 175.0716, 191.0724, 251.0936, 219.0607, 245.0854 |
| 63^a^ | 1E,6E)-1-(4-hydroxyphenyl)-7-phenylhepta-1,6-diene-3,5-dione | C_19_H_17_O_3_ | 293.1178 | 293.1196 | 6.14 | 16.97 | 77.0377, 93.0324, 119.0493, 145.0628, 161.0587, 131.0488, 215.0717 |
| 64^a^ | (1E,6E)-1,7-bis(4-hydroxyphenyl)hepta-1,6-diene-3,5-dione | C_19_H_17_O_4_ | 309.1127 | 309.1119 | -2.59 | 14.74 | 93.0304, 215.0657, 119.0493, 189.0523, 161.0587, 147.0439 |
| 65^*a^ | Bisdemethoxycurcumin | C_19_H_17_O_4_ | 309.1127 | 309.1119 | -2.59 | 19.52 | 93.0343, 215.0717, 119.0493, 189.0552, 161.0587, 147.0439 |
| 66^a^ | (1E,6E)-1-(4-hydroxy-3-methoxyphenyl)-7-phenylhepta-1,6-diene-3,5-dione | C_20_H_19_O_4_ | 323.1283 | 323.1261 | -6.81 | 17.97 | 177.0539, 145.0628, 131.0512, 191.0724, 103.0534, 149.0583, 77.0395 |
| 67^a^ | (1E,6E)-1-(4-hydroxy-3-methoxyphenyl)-7-(4-hydroxyphenyl)hepta-1,6-diene-3,5-dione | C_20_H_19_O_5_ | 339.1232 | 339.1259 | 7.96 | 14.95 | 123.0411, 215.0657, 149.0583, 177.0539, 161.0612, 147.0439, 119.0493, 93.0304 |
| 68^*a^ | Demethoxycurcumin | C_20_H_19_O_5_ | 339.1232 | 339.1222 | -2.95 | 19.71 | 123.0411, 215.0717, 149.0583, 189.0552, 177.0539, 161.0587, 147.0439, 191.0668, 119.0493, 93.0324, 245.0790 |
| 69^a^ | (1E,6E)-1-(3,4-dihydroxyphenyl)-7-(4-hydroxy-3-methoxyphenyl)hepta-1,6-diene-3,5-dione | C_20_H_19_O_6_ | 355.1182 | 355.1189 | 1.97 | 17.97 | 149.0608, 205.0502, 177.0539, 135.0451, 109.0294 |
| 70^a^ | (1E,6E)-1,7-bis(4-hydroxy-3-methoxyphenyl)hepta-1,6-diene-3,5-dione | C_21_H_21_O_6_ | 369.1338 | 369.1333 | -1.35 | 15.17 | 245.0822, 123.0411, 149.0583, 177.0539, 191.0668 |
| 71^*a^ | curcumin | C_21_H_21_O_6_ | 369.1338 | 369.1333 | -1.35 | 19.86 | 191.0696, 177.0539, 149.0583, 123.0411 |
| 72^a^ | (1E,6E)-1-(4-hydroxy-3,5-dimethoxyphenyl)-7-(4-methoxyphenyl)hepta-1,6-diene-3,5-dione | C_22_H_23_O_6_ | 383.1495 | 383.1465 | -7.83 | 11.42 | 179.0720, 221.0856, 133.0619, 249.0806, 107.0511, 275.0934 |
| 73^a^ | (1E,4E,6E)-7-(4-hydroxy-3-methoxyphenyl)-1-(4-hydroxyphenyl)hepta-1,4,6-trien-3-one | C_20_H_19_O_4_ | 323.1283 | 323.1298 | 4.64 | 17.16 | 123.0479, 199.0752, 149.0583, 175.0743, 119.0470 |
| 74^a^ | (1E,4E,6E)-7-(4-hydroxy-3,5-dimethoxyphenyl)-1-phenylhepta-1,4,6-trien-3-one | C_21_H_21_O_4_ | 337.1440 | 337.1429 | -3.26 | 28.83 | 175.0743, 161.0587, 149.0583, 133.0619, 203.0681, 107.0490 |
| 75^a^ | (1E,4E,6E)-7-(4-hydroxy-3-methoxyphenyl)-1-(4-methoxyphenyl)hepta-1,4,6-trien-3-one | C_21_H_21_O_4_ | 337.1440 | 337.1429 | -3.26 | 12.62 | 153.0524, 157.0685, 131.0488, 205.0853, 103.0555, 77.0377 |
| 76^a^ | (1E,4E,6E)-1-(4-methoxyphenyl)-7-(2,3,4,5-tetramethoxyphenyl)hepta-1,4,6-trien-3-one | C_24_H_27_O_6_ | 411.1808 | 411.1797 | -2.68 | 21.34 | 161.0587, 133.0643, 197.0821, 213.0955, 187.0766, 183.0649, 167.0690, 153.0549 |
| 77^a^ | (1E,4E,6E)-1-(3,4-dimethoxyphenyl)-7-(3,4,5-trimethoxyphenyl)hepta-1,4,6-trien-3-one | C_24_H_27_O_6_ | 411.1808 | 411.1797 | -2.68 | 19.60 | 217.0873, 191.0752, 137.0597 |
| 78^a^ | (E)-2-(3,4-dihydroxybenzylidene)-5-((E)-4-hydroxystyryl)furan-3(2H)-one | C_19_H_15_O_5_ | 323.0919 | 323.0930 | 3.40 | 19.45 | 119.0493, 161.0587, 109.0294, 213.0537 |
| 79^a^ | (E)-2-benzylidene-5-((E)-3,4-dimethoxystyryl)furan-3(2H)-one | C_21_H_19_O_4_ | 335.1283 | 335.1281 | -0.60 | 25.41 | 137.0597, 77.0395, 257.0795 |
| 80^a^ | (E)-5-((E)-3,4-dimethoxystyryl)-2-(3,4,5-trihydroxybenzylidene)furan-3(2H)-one | C_21_H_19_O_7_ | 383.1131 | 383.1104 | -7.05 | 15.79 | 191.0668, 177.0185, 205.0824, 163.0777, 125.0239 |
| 81^a^ | (E)-2-(4-methoxybenzylidene)-5-((E)-3,4,5-trimethoxystyryl)furan-3(2H)-one | C_23_H_23_O_6_ | 395.1495 | 395.1476 | -4.81 | 21.22 | 227.0683, 221.0826 |
| 82^a^ | (E)-5-((E)-4,5-dihydroxy-2,3-dimethoxystyryl)-2-(3,4-dihydroxy-5-methoxybenzylidene)furan-3(2H)-one | C_22_H_21_O_9_ | 429.1186 | 429.1188 | 0.47 | 16.69 | 169.0508, 195.0618, 237.0752, 193.0492 |
| 83^b^ | Liquiritigenin | C_15_H_12_O_4_ | 257.0814 | 257.0797 | -6.6 | 8.43 | 229.0881, 137.0219, 121.0281 |
| 84^b^ | Isoliquiritin | C_21_H_22_O_9_ | 419.1342 | 419.1310 | -7.6 | 12.10 | 257.0795, 147.0439, 119.0493 |
| 85^b^ | Uralenol | C20H16O7 | 369.0974 | 369.0979 | 1.4 | 24.29 | 341.1035, 153.0217 |
| 86^b^ | Daidzein | C_15_H_10_O_4_ | 255.0657 | 255.0645 | -4.7 | 12.81 | 163.0403, 139.0415, 119.0484 |
| 87^b^ | 2',7-Dihydroxy-4'-methoxyisoflavone | C_16_H_12_O_5_ | 285.0763 | 285.0754 | -3.2 | 27.86 | 163.0377, 139.0366, 149.0582 |
| 88^b^ | Liquiritinapioside | C_26_H_30_O_13_ | 551.1765 | 551.1725 | -7.3 | 11.54 | 419.1310, 257.0764, 229.0850, 137.0219, 121.0258 |
| 89^b^ | 7-O-Methylluteone | C_21_H_18_O_6_ | 367.1182 | 367.1191 | 2.5 | 5.36 | 259.0983, 135.0433 |
| 90^b^ | Licochalcone A | C_21_H_22_O_4_ | 339.1596 | 339.1599 | 0.88 | 22.46 | 325.1411, 231.1043, 257.0828 |
| 91^b^ | Eurycarpin A | C_20_H_16_O_5_ | 337.1076 | 337.1053 | -6.8 | 20.48 | 163.0377, 139.0415, 201.0899 |
| 92^b^ | Angustone A | C_25_H_22_O_6_ | 419.1495 | 419.1477 | -4.3 | 13.40 | 245.0830, 221.0785, 201.0899 |
| 93^b^ | Licochalcone B | C_16_H_14_O_5_ | 287.0919 | 287.0898 | -7.3 | 12.85 | 273.0808, 179.0309 |
| 94^b^ | Abyssinone Ⅱ | C_21_H_20_O_4_ | 337.1440 | 337.1429 | -3.3 | 17.97 | 309.1479, 137.0219, 121.0281 |
| 95^b^ | (2R,3R)-3,4',7-trihydroxy-3'-prenylflavane | C_21_H_20_O_5_ | 353.1389 | 353.1357 | -9.1 | 17.97 | 325.1414, 137.0219, 121.0281 |
| 96^b^ | Paratocarpin L | C_26_H_26_O_5_ | 433.2015 | 433.2018 | 0.7 | 34.81 | 405.2137, 233.0854, 217.0864 |
| 97^b^ | 3-Hydroxyglabrol | C_27_H_28_O_5_ | 433.2015 | 433.2022 | 1.6 | 26.69 | 405.2055, 201.0928 |
| 98^b^ | Isoangustone A | C_25_H_26_O_6_ | 423.1808 | 423.1794 | -3.3 | 27.86 | 247.0958, 223.0912, 203.1059 |
| 99^b^ | 7,4'-Dihydroxy-3'-methoxyisoflavan | C_16_H_16_O_4_ | 273.1127 | 273.1107 | -7.3 | 19.09 | 275.0934, 149.0583, 123.0411 |
| 100^b^ | Licochalcone I | C_22_H_22_O_5_ | 367.1545 | 367.1544 | -0.30 | 23.48 | 353.1329, 179.0364 |
| 101^b^ | Licochalcone K | C_21_H_22_O_5_ | 355.1545 | 355.1536 | -2.5 | 21.19 | 341.1418, 163.0412, 135.0451 |
| 102^b^ | 5-(1,1-dimethylallyl)-3,4,4'-trihydroxy-2-methoxychalcone | C_21_H_22_O_5_ | 355.1545 | 355.1536 | -2.5 | 19.76 | 341.1418, 179.0336, 151.0412 |
| 103^b^ | Isoviolanthin | C_27_H_30_O_14_ | 579.1714 | 579.1764 | 8.6 | 10.05 | 433.1055, 417.1187, 271.0589, 243.0648, 153.0195, 137.0214 |
| 104^b^ | Iso-liquiritigenin | C_15_H_12_O_4_ | 257.0814 | 257.0830 | 6.2 | 8.69 | 147.0439, 119.0493 |
| 105^b^ | Daidzin | C_21_H_20_O_9_ | 417.1186 | 417.1183 | -0.7 | 8.74 | 255.0658, 163.0377, 139.0366, 119.0484 |
| 106^b^ | Licorisoflavan A | C_27_H_34_O_5_ | 439.2484 | 439.2503 | 4.3 | 13.09 | 425.2369, 411.2213 |
| 107^*b^ | Glycyrrhizin | C_42_H_62_O_16_ | 823.4116 | 823.4088 | -3.4 | 22.65 | 647.3810, 471.3462, 453.3364 |
| 108^b^ | 18β‐Glycyrrhetinic acid | C_30_H_46_O_4_ | 471.3474 | 471.3518 | 9.3 | 22.65 | 453.3364 |
| 109^b^ | Glycyrrhetic acid | C_36_H_54_O_10_ | 647.3795 | 647.3781 | -2.2 | 22.65 | 471.3462, 453.3364 |
| 110^b^ | Licoricesaponin G2 | C_42_H_62_O_17_ | 839.4065 | 839.4108 | 5.1 | 21.26 | 663.3739, 487.3418, 469.3312 |
| 111^b^ | Licochalcone D | C_21_H_22_O_5_ | 355.1545 | 355.1536 | -2.5 | 22.81 | 341.1380, 179.0309, 135.0451 |
| 112^b^ | Licoricesaponin A3 | C_48_H_72_O_21_ | 985.4644 | 985.4646 | 0.2 | 18.74 | 809.4288, 647.3810, 633.3985, 471.3462, 453.3364 |
| 113^b^ | 22-Acetoxyl-glycyrrhizin | C_44_H_64_O_18_ | 881.4171 | 881.4197 | 2.9 | 18.36 | 705.3755, 529.3527, 511.3433 |
| 114^b^ | Licochalcone C | C_21_H_22_O_4_ | 339.1596 | 339.1599 | 0.9 | 22.31 | 325.1411, 231.0981, 203.1031 |
| 115^b^ | Echinayin | C_16_H_14_O_4_ | 271.0970 | 271.0955 | -5.5 | 14.12 | 257.0795, 163.0385, 135.0427 |
| 116^b^ | Ononin | C_22_H_22_O_9_ | 431.1342 | 431.1347 | 1.2 | 11.85 | 269.0810, 137.0219 |
| 117^b^ | Glycyuralin F | C_20_H_20_O_6_ | 357.1338 | 357.1308 | -8.4 | 34.81 | 249.1104, 225.1161, 135.0457 |
| 118^b^ | 6,8-Diprenylgenistein | C_25_H_26_O_5_ | 407.1858 | 407.1852 | -1.5 | 36.55 | 315.1604, 275.1640, 119.0462 |
| 119^b^ | Calycosin | C_16_H_14_O_5_ | 287.0919 | 287.0898 | -7.3 | 12.04 | 257.0795, 229.0803, 199.0781 |
| 120^b^ | Echinatin | C_16_H_14_O_4_ | 271.0970 | 271.0955 | -5.5 | 15.42 | 257.0828, 163.0359 |
| 121^b^ | Licoflavone A | C_20_H_18_O_4_ | 323.1283 | 323.1261 | -6.8 | 21.07 | 255.0622, 227.0714, 137.0214 |
| 122^b^ | Formononetin | C_16_H_12_O_4_ | 269.0814 | 269.0810 | -1.5 | 16.41 | 163.0403, 139.0415, 133.0626 |
| 123^b^ | Afromosin | C_17_H_14_O_5_ | 299.0919 | 299.0917 | -0.70 | 23.40 | 193.0496, 169.0470, 133.0650 |
| 124^b^ | Pallidiflorin | C_16_H_12_O_4_ | 269.0814 | 269.0810 | -1.5 | 11.85 | 163.0377, 139.0366, 133.0673 |
| 125^b^ | Methoxychalcone | C_16_H_14_O_4_ | 271.0970 | 271.0989 | 7.0 | 16.66 | 257.0795, 147.0439, 119.0515 |
| 126^b^ | Vestitol | C_16_H_16_O_4_ | 273.1127 | 273.1107 | -7.3 | 17.19 | 149.0583, 123.0456 |
| 127^b^ | 6″-O-Acetylanonin | C_24_H_24_O_10_ | 473.1448 | 473.1440 | -1.7 | 9.74 | 431.1347, 269.0810, 147.0464, 123.0423, 133.0650 |
| 128^b^ | 6″-O-Acetylwistin | C_25_H_26_O_11_ | 503.1553 | 503.1572 | 3.8 | 21.57 | 461.1449, 193.0467, 169.0470, 133.0650 |
| 129^b^ | Kanzonol C | C_25_H_28_O_4_ | 393.2066 | 393.2097 | 7.9 | 6.83 | 147.0414 |
| 130^b^ | 2'-Hydroxyisolupalbigenin | C_25_H_26_O_5_ | 407.1858 | 407.1852 | -1.5 | 38.17 | 231.0996, 207.1034, 203.1059 |
| 131^b^ | 6,8-diprenyl-apigenin | C_25_H_26_O_5_ | 407.1858 | 407.1852 | -1.5 | 26.43 | 379.1977, 339.1225, 311.1254, 221.0826 |
| 132^b^ | Gancaonin Q | C_25_H_26_O_5_ | 407.1858 | 407.1852 | -1.5 | 26.59 | 379.1952 |
| 133^b^ | Licoflavone B | C_25_H_26_O_4_ | 391.1909 | 391.1914 | 1.3 | 28.02 | 323.1294, 363.1986, 205.0883, 189.0889 |
| 134^b^ | Glyasperin C | C_21_H_24_O_5_ | 357.1702 | 357.1695 | -2.0 | 19.60 | 207.1007, 165.0542, 139.0384 |
| 135^b^ | Glyurallin B | C_25_H_26_O_6_ | 423.1808 | 423.1794 | -3.3 | 27.52 | 247.0990, 203.1059 |
| 136^b^ | Glycycoumarin | C_21_H_22_O_6_ | 371.1495 | 371.1490 | -1.3 | 23.40 | 357.1308, 353.1357 |
| 137^*b^ | Liquiritin | C_21_H_22_O_9_ | 419.1342 | 419.1310 | -7.6 | 11.60 | 257.0797, 229.0881, 137.0243, |
| 138^b^ | Topazolin | C_21_H_18_O_6_ | 367.1182 | 367.1191 | 2.5 | 18.98 | 325.1042, 301.0718, 297.1115, 287.0565, 259.0652, 273.0741, 231.0669, 137.0238 |
| 139^c^ | Chlorogenic acid methyl ester | C_17_H_20_O_9_ | 369.1186 | 369.1156 | -8.13 | 2.69 | 351.1053, 163.0359, 145.0259 |
| 140^c^ | Cryptochlorogenic acid methyl ester | C_17_H_20_O_9_ | 369.1186 | 369.1156 | -8.13 | 2.54 | 351.1053, 163.0385, 145.0283 |
| 141^c^ | Linarin | C_28_H_32_O_13_ | 577.1921 | 577.1913 | -1.39 | 11.42 | 431.1358, 285.0732, 153.0169 |
| 142^c^ | Arcapillin | C_18_H_16_O_8_ | 361.0923 | 361.0941 | 4.98 | 5.83 | 347.0714, 197.0476 |
| 143^c^ | Cirsimaritin | C_17_H_14_O_6_ | 315.0869 | 315.0896 | 8.57 | 17.47 | 301.0718, 287.0981, 197.0476 |
| 144^c^ | Scoparone | C_11_H_10_O_4_ | 207.0657 | 207.0651 | -2.90 | 16.91 | 192.0468, 164.0461, 136.0506, 179.0747 |
| 145^c^ | Scopoletin | C_10_H_8_O_4_ | 193.0501 | 193.0496 | -2.59 | 9.05 | 165.0542, 137.0549 |
| 146^c^ | Isofraxidin 7-O-β-D-glucopyranoside | C_17_H_20_O_10_ | 385.1135 | 385.1158 | 5.97 | 37.05 | 223.0618, 208.0383, 195.0676, 179.0665 |
| 147^c^ | Neochlorogenic acid | C_16_H_18_O_9_ | 355.1029 | 355.1035 | 1.69 | 4.71 | 309.1000, 163.0385, 145.0283 |
| 148^c^ | Umbelliferone | C_9_H_6_O_3_ | 163.0395 | 163.0403 | 4.91 | 25.53 | 135.0427, 119.0493, 107.0533 |
| 149^c^ | Isoscopoletin | C_10_H_8_O_4_ | 193.0501 | 193.0496 | -2.59 | 16.91 | 178.0258, 150.0267, 121.0281, 165.0533, 149.0582, 137.0627 |
| 150^c^ | 7-Methoxy coumarin | C_10_H_8_O_3_ | 177.0552 | 177.0551 | -0.56 | 10.61 | 162.0341, 134.0386, 149.0558, 121.0651, 133.0643 |
| 151^c^ | Chlorogenic acid | C_16_H_18_O_9_ | 355.1029 | 355.0996 | -9.29 | 4.28 | 309.1000, 163.0385, 145.0259 |
| 152^c^ | Isofraxidin 7-O-(6’-O-p-coumaroyl)-𝛽-glucopyranoside | C_26_H_26_O_12_ | 531.1503 | 531.1510 | 1.32 | 8.69 | 223.0588, 208.0383, 180.0400, 152.0414, 195.0647, 167.0743, 179.0720 |
| 153^*c^ | Caffeic acid | C_9_H_8_O_4_ | 181.0501 | 181.0501 | 0.00 | 25.57 | 163.0377 |
| 154^d^ | Schisandrin | C_24_H_32_O_7_ | 433.2226 | 433.2193 | -7.62 | 17.97 | 455.2076, 415.2108, 385.1989, 361.1617 |
| 155^d^ | (7S,8R)-Urolignoside | C_25_H_32_O_11_ | 509.2023 | 509.2022 | -0.20 | 22.12 | 531.1953, 347.1476 |
| 156^d^ | Gomisin D | C_28_H_34_O_10_ | 531.2230 | 531.2217 | -2.45 | 19.21 | 485.2156, 401.1577, 383.1492, 351.1245, 352.1299, 368.1254, 353.1329 |
| 157^d^ | Icariside E4 | C_26_H_34_O_10_ | 507.2230 | 507.2265 | 6.90 | 27.21 | 347.1476 |
| 158^d^ | 3-O-β-d-Glucopyranosyloxymethyl-2-(4-hydroxy-3-methoxyphenyl)-5-(3-hydroxypropyl)-7-methoxy-dihydrobenzofuran | C_26_H_34_O_11_ | 523.2179 | 523.2151 | -5.35 | 27.21 | 347.1476 |
| 159^d^ | (+)-Pinoresinol | C_20_H_22_O_6_ | 359.1495 | 359.1485 | -2.78 | 17.97 | 341.1380, 311.1254, 217.0842, 323.1294, 187.0766, 137.0597 |
| 160^d^ | Prestegane B | C_20_H_22_O_6_ | 359.1495 | 359.1524 | 8.07 | 20.69 | 341.1342, 323.1257, 291.0996, 263.1060, 137.0573 |
| 161^d^ | Gomisin A | C_23_H_28_O_7_ | 417.1913 | 417.1893 | -4.79 | 19.95 | 439.1743, 399.1805, 384.1553, 368.1608 |
| 162^d^ | (7S,8R,7’R,8’R)-7-(3,4-methylenedioxyphenyl)-8,8’-dimethyl-8’-hydroxyl-7’-methoxyl-7’-(3’,4’-methylenedioxyphenyl)-tetrahydrofuran | C_21_H_22_O_7_ | 387.1444 | 387.1424 | -5.17 | 34.10 | 163.0725, 181.0491, 209.0816, 151.0412, 237.1161, 149.0258 |
| 163^d^ | (+)-Zuihonin A | C_20_H_20_O_5_ | 341.1389 | 341.1375 | -4.10 | 29.41 | 163.0751, 179.0692, 191.1035 |
| 164^d^ | (-)-Zuihonin C | C_21_H_24_O_5_ | 357.1702 | 357.1695 | -1.96 | 18.00 | 207.1390, 195.1019, 163.0777, 191.1092, 150.0243 |
| 165^d^ | 14-Tigloylschinlignan D | C_27_H_32_O_8_ | 485.2175 | 485.2161 | -2.89 | 19.17 | 452.1825, 436.1902 |
| 166^d^ | Schibitubin A | C_20_H_24_O_4_ | 329.1753 | 329.1759 | 1.82 | 30.62 | 311.1578, 135.0427, 121.0628 |
| 167^d^ | Schibitubin D | C_22_H_26_O_6_ | 387.1808 | 387.1786 | -5.68 | 24.83 | 327.1553, 137.0597, 135.0475 |
| 168^d^ | Schisantherin B | C_28_H_34_O_9_ | 515.2281 | 515.2276 | -0.97 | 25.14 | 537.2070 |
| 169^d^ | Schibitubin E | C_22_H_26_O_6_ | 387.1808 | 387.1827 | 4.91 | 24.98 | 409.1655, 135.0427, 137.0597 |
| 170^d^ | Schibitubin F | C_22_H_28_O_6_ | 389.1964 | 389.1946 | -4.62 | 19.60 | 411.1757, 137.0597 |
| 171^d^ | Schibitubin H | C_22_H_24_O_7_ | 401.1600 | 401.1582 | -4.49 | 19.21 | 179.0692, 163.0777, 191.1035, 149.0233 |
| 172^d^ | (−)-Futokadsurin A | C_21_H_26_O_5_ | 359.1858 | 359.1873 | 4.18 | 27.67 | 165.0936, 195.1047, 167.0690, 181.0877, 207.1361 |
| 173^d^ | Deoxyschisandrin | C_24_H_32_O_6_ | 417.2277 | 417.2269 | -1.92 | 25.69 | 401.1946, 373.1922, 361.1617, 347.1476, 316.1303, 301.1073 |
| 174^d^ | (+)-Zuonin A | C_20_H_20_O_5_ | 341.1389 | 341.1375 | -4.10 | 23.40 | 163.0751, 179.0692, 191.1035 |
| 175^d^ | Kadlongirin C | C_23_H_28_O_7_ | 417.1913 | 417.1877 | -8.63 | 34.22 | 209.1201, 209.0786, 181.0491, 225.1113, 193.0862, 197.0792, 195.0647 |
| 176^d^ | Schinlignans C | C_28_H_36_O_8_ | 501.2488 | 501.2495 | 1.40 | 20.64 | 523.2307, 483.2341, 468.2102, 413.1533, 367.1170 |
| 177^d^ | Schinlignans E | C_27_H_32_O_8_ | 485.2175 | 485.2206 | 6.39 | 19.02 | 452.1803, 436.1931 |
| 178^d^ | Schisandrin B | C_23_H_28_O_6_ | 401.1964 | 401.1951 | -3.24 | 27.86 | 423.1758, 386.1715, 370.1779, 302.1096, 287.0877, 272.1010 |
| 179^d^ | Schinlignans G | C_28_H_36_O_7_ | 485.2539 | 485.2522 | -3.50 | 27.21 | 507.2375, 454.2345 |
| 180^d^ | Angeloylgomisin H | C_28_H_36_O_8_ | 501.2488 | 501.2495 | 1.40 | 19.83 | 523.2307, 483.2386, 468.2147, 413.1575, 367.1170 |
| 181^d^ | (7S, 8S, 7′R, 8′S)-7-methoxyl-7-(3,4-methylenedioxy-phenyl)-8-hydroxyl-8′-methyl-7′-(3′,4′,5′-trimethoxyphenyl)-tetrahydrofuran | C_23_H_28_O_8_ | 433.1862 | 433.1852 | -2.31 | 31.81 | 225.0744, 237.1476, 181.0519, 195.0676, 149.0258 |
| 182^d^ | (7S, 8S, 7′R, 8′S)-7-methoxyl-7-(3,4-methylenedioxyphenyl)-8-hydroxyl-8′-methyl-7′-(3′,4′-methylenedioxyphenyl)-tetrahydrofuran | C_21_H_22_O_5_ | 355.1545 | 355.1574 | 8.17 | 25.69 | 119.0493, 237.1161, 117.0371, 149.0208 |
| 183^d^ | (7S, 8S, 7′R, 8′S)-(7, 8-trans-8, 8′-trans-7′, 8′-trans)-7-methoxyl-7-(3,4- methylenedioxy-phenyl)-8 methyl -8′-methyl-7′-(3′,4′-dimethoxyphenyl)-tetrahydrofuran | C_22_H_26_O_6_ | 387.1808 | 387.1827 | 4.91 | 35.03 | 179.1076, 181.0495, 195.1047, 193.0890, 167.0690, 165.0542, 149.0208 |
| 184^d^ | (7S, 8S, 7′R, 8′S)-7-methoxyl-7-(3,4,5-trimethoxyphenyl)-8methyl -8′-methyl-7′-(3′,4′- methylenedioxyphenyl)-tetrahydrofuran | C_23_H_28_O_8_ | 433.1862 | 433.1852 | -2.31 | 32.05 | 163.0751, 271.1128, 191.1035, 283.1552 |
| 185^d^ | Schisandrin C | C_22_H_24_O_6_ | 385.1651 | 385.1639 | -3.12 | 29.26 | 407.1479, 354.1451, 286.0795, 271.0589, 256.0731 |
| 186^d^ | (7S, 8S, 7′R, 8′S)-7-(3,4- methylenedioxyphenyl)-8-methyl -8′-methyl-7′-(3′,4′- dihydroxy-phenyl)-tetrahydrofuran | C_19_H_20_O_5_ | 329.1389 | 329.1388 | -0.30 | 17.93 | 151.0764, 163.0725, 191.1092, 149.0233 |
| 187^d^ | Schiglaucin B | C_22_H_26_O_5_ | 371.1858 | 371.1826 | -8.62 | 27.86 | 209.1171, 221.1161, 133.0666 |
| 188^d^ | Schisantherin D | C_29_H_28_O_9_ | 521.1812 | 521.1763 | -9.40 | 23.21 | 503.1621, 399.1477, 369.1392 |
| 189^d^ | Schisantherin A | C_30_H_32_O_9_ | 537.2125 | 537.2096 | -5.40 | 23.40 | 415.1732, 299.0901, 371.1511, 356.1273, 340.1333, 325.1078, 312.0971, 282.0853, 267.1011, 255.0622 |
| 190^d^ | Methylgomisin O | C_24_H_30_O_7_ | 431.2070 | 431.2070 | 0.00 | 20.88 | 453.1883, 416.1788 |
| 191^d^ | Methylisogomisin O | C_24_H_30_O_7_ | 431.2070 | 431.2077 | 1.62 | 20.48 | 453.1883, 416.1788, 387.1815, 370.1779 |
| 192^d^ | 7(18)-Dehydroschisandro A | C_24_H_30_O_6_ | 415.2121 | 415.2105 | -3.85 | 17.97 | 400.1869, 385.1949 |
| 193^d^ | Rubschisantherin | C_25_H_30_O_8_ | 459.2019 | 459.2028 | 1.96 | 17.96 | 401.1906, 386.1715, 370.1740, 302.1131, 287.0877, 272.1044 |
| 194^d^ | Schisphenlignans F | C_22_H_30_O_6_ | 391.2121 | 391.2117 | -1.02 | 22.09 | 413.1949, 167.0690 |
| 195^d^ | Schilignan F | C_26_H_32_O_12_ | 537.1972 | 537.2013 | 7.63 | 20.85 | 359.1460, 341.1380, 323.1257, 311.1254, 187.0766, 137.0621 |
| 196^d^ | Isomassonianoside B | C_25_H_32_O_10_ | 493.2074 | 493.2093 | 3.85 | 24.98 | 475.1972, 347.1438, 329.1312 |
| 197^d^ | Schisanlactone B | C_30_H_42_O_4_ | 467.3161 | 467.3136 | -5.35 | 38.94 | 327.2296 |
| 198^d^ | Lancifodilactone D | C_29_H_34_O_9_ | 527.2281 | 527.2250 | -5.88 | 37.05 | 483.2393, 469.2262 |
| 199^d^ | Micrandilactone A | C_29_H_36_O_12_ | 577.2285 | 577.2291 | 1.04 | 37.08 | 559.2152, 533.2328, 499.1899, 471.2008 |
| 200^d^ | Micrandilactone D | C_29_H_36_O_11_ | 561.2336 | 561.2366 | 5.35 | 39.87 | 543.2216, 517.2502, 483.1988 |
| 201^d^ | Henridilactone D | C_29_H_36_O_10_ | 545.2387 | 545.2361 | -4.77 | 37.05 | 527.2203, 485.2161, 439.2157 |
| 202^d^ | Heteroclitalactone F | C_31_H_46_O_4_ | 483.3474 | 483.3517 | 8.90 | 24.20 | 469.3237, 329.2539 |
| 203^d^ | Henridilactone B | C_29_H_34_O_11_ | 559.2179 | 559.2200 | 3.76 | 34.75 | 499.1899, 471.2097 |
| 204^d^ | Schisanlactone I | C_32_H_50_O_5_ | 515.3736 | 515.3716 | -3.88 | 23.02 | 487.3461, 347.2536 |
| 205^d^ | Schiglausin P | C_33_H_50_O_7_ | 559.3635 | 559.3652 | 3.04 | 37.67 | 485.3288, 345.2355 |
| 206^d^ | Kadsuphilactone B | C_30_H_42_O_5_ | 483.3110 | 483.3112 | 0.41 | 14.28 | 465.2319, 327.2333 |
| 207^e^ | Catalpol | C_15_H_22_O_10_ | 385.1111** | 385.1077 | -8.83 | 1.02 | 201.0783, 183.0657, 151.0360, 133.0272 |
| 208^e^ | Ajugol | C_15_H_24_O_9_ | 371.1318** | 371.1293 | -6.74 | 1.17 | 187.0997, 169.0842 |
| 209^e^ | Rehmannioside D | C_27_H_42_O_20_ | 709.2167** | 709.2172 | 0.71 | 0.98 | 669.2244, 527.1592, 365.1065, 203.0505 |
| 210^e^ | Rehmannioside A | C_21_H_32_O_15_ | 547.1639** | 547.1635 | -0.73 | 0.98 | 507.1725, 365.1065, 203.0505 |

This table has been published in Shi Q, He J, Chen G, Jinlin Xu, Zhaoxiang Zeng, Xueyan Zhao, et al. The chemical composition of Diwu YangGan capsule and its potential inhibitory roles on hepatocellular carcinoma by microarray-based transcriptomics. J Tradit Complement Med. 2023;14(4):381-390. Published 2023 Dec 25. doi:10.1016/j.jtcme.2023.12.002

# Supplementary Figure

## Supplementary Figure 1


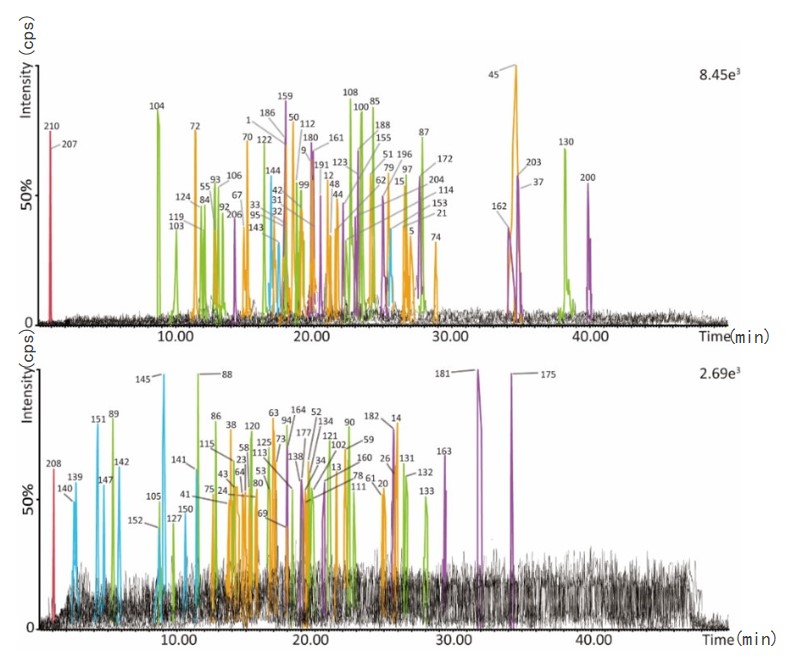


EIC diagrams of DWYG compounds based on UPLC-QTOF-MS/MS.

This figure has been published in Shi Q, He J, Chen G, Jinlin Xu, Zhaoxiang Zeng, Xueyan Zhao, et al. The chemical composition of Diwu YangGan capsule and its potential inhibitory roles on hepatocellular carcinoma by microarray-based transcriptomics. J Tradit Complement Med. 2023;14(4):381-390. Published 2023 Dec 25. doi:10.1016/j.jtcme.2023.12.002
